# Leaf chamber experiments on sunflowers indicate a temperature-dependent compensation point of carbonyl sulfide

**DOI:** 10.12688/openreseurope.20235.3

**Published:** 2026-04-10

**Authors:** Ara Cho, Linda M.J. Kooijmans, Steven M. Driever, Maarten Wassenaar, Gerbrand Koren, Sophie L. Baartman, Leon Mossink, Maarten C. Krol

**Affiliations:** 1 Meteorology and Air Quality, Wageningen University & Research, Wageningen, Gelderland, The Netherlands; 2 Centre for Crop Systems Analysis, Wageningen University & Research, Wageningen, Gelderland, The Netherlands; 3 Horticulture and Product Physiology, Wageningen University & Research, Wageningen, Gelderland, The Netherlands; 4 Copernicus Institute of Sustainable Development, Utrecht University, Utrecht, Utrecht, The Netherlands; 5 Institute for Marine and Atmospheric Research, Utrecht University, Utrecht, The Netherlands

**Keywords:** Leaf chamber experiment, Leaf conductance model, Carbonyl sulfide, Photosynthesis, Leaf relative uptake, Stomatal conductance, Compensation point

## Abstract

**Background:**

Carbonyl Sulfide (COS) is a potential tracer for estimating gross primary productivity (GPP), due to its co-uptake with CO _2_ in leaves and the assumed absence of re-emission. However, the effectiveness of COS as a GPP tracer depends on understanding the differential responses of COS and CO _2_ uptake to environmental factors such as temperature and humidity. Methods We conducted three sets of leaf gas exchange experiments on sunflower leaves. In each experiment, we varied only one environmental factor: COS mole fraction (at two temperatures), humidity, or temperature. During the experiments, COS and CO _2_ fluxes were measured, and the data were used to optimize a leaf conductance model.

**Results:**

We identified the existence of a COS compensation point, which increases with higher temperatures, suggesting potential emissions at higher temperatures when atmospheric COS concentrations are low. Our gas exchange measurements detected a COS compensation point of 58.9 ± 52.4 pmol mol ^−1^ at 20°C and 139.9 ± 26.0 pmol mol ^−1^ at 25°C. As vapor pressure deficit increased and stomatal conductance decreased, we observed that COS leaf uptake decreased more rapidly than CO _2_ assimilation. Consequently, the leaf relative uptake ratio (LRU) of COS to CO _2_ also decreased when stomatal conductance decreased. The optimized conductance model indicated that the optimum temperature for COS and CO _2_ enzymatic uptake was around 35°. However, the maximum net deposition velocity for COS lies between 20 and 25°, due to its temperature-dependent compensation point.

## Plain language summary

Carbonyl sulfide (COS) is a trace gas found in the atmosphere that plants take up in a way similar to carbon dioxide (CO _2_) during photosynthesis. Because of this shared pathway, COS has been widely used to estimate how much CO _2_ plants absorb from the atmosphere—a process known as gross primary production (GPP). In our study, we conducted experiments using sunflower leaves in controlled chambers and found that COS and CO _2_ respond differently to changes in temperature and humidity. Surprisingly, we also observed that sunflower leaves may release COS back into the atmosphere when COS levels are low and temperatures are high. This finding challenges the long-standing assumption that plants only absorb COS and do not emit it. Our research highlights that understanding how COS behaves in plants under different environmental conditions is crucial for using it as a reliable tool to estimate how much CO _2_ is absorbed by vegetation.

## 1. Introduction

Gross primary productivity (GPP) quantifies the largest terrestrial CO _2_ uptake flux in the global carbon cycle. However, when measuring the net ecosystem exchange (NEE) of CO _2_, separating the GPP signal from respiration is challenging ( Reichstein *et al.*, 2005; Wohlfahrt & Gu, 2015). To address this limitation, Carbonyl Sulfide (COS) is an atmospheric trace gas that has been identified as a promising tracer for GPP and several studies have explored its utility extensively ( Asaf *et al.*, 2013; Campbell *et al.*, 2008; Kohonen *et al.*, 2022; Montzka *et al.*, 2007). COS primarily originates from anthropogenic sources and oceans, while vegetation predominantly acts as its sink ( Berry *et al.*, 2013; Kettle *et al.*, 2002).

The application of COS to estimate GPP has been suggested because COS uptake by leaves proceeds through a stomatal pathway similar to uptake of CO _2_. Importantly, COS is assumed not to be released by plants due to its irreversible hydrolysis catalyzed by carbonic anhydrase (CA) ( Notni *et al.*, 2007; Protoschill-Krebs *et al.*, 1996; Stimler *et al.*, 2010). With this advantage of COS, the leaf relative uptake ratio (LRU) of deposition velocities of COS and CO _2_ has been introduced to scaling COS leaf uptake to photosynthesis ( Campbell *et al.*, 2008; Sandoval-Soto *et al.*, 2005) (a detailed LRU description is provided in Section 2.1.2). However, previous studies have emphasized that LRU responds differently to environmental factors such as leaf temperature ( *T _leaf_*) and vapor pressure deficit (VPD), implying that it is not constant under varying conditions ( Cochavi *et al.*, 2021; Kooijmans *et al.*, 2019; Stimler *et al.*, 2010; Sun *et al.*, 2018; Wohlfahrt *et al.*, 2018).

Unlike photosynthesis, COS uptake by CA remains unaffected by light, rendering it a robust proxy for stomatal conductance to water vapor ( *g _sw_*). Accordingly, recent studies have used ecosystem-level COS exchange as a proxy for *g _sw_*, highlighting differences between stomatal and biochemical contributions to CO _2_ uptake ( Cochavi *et al.*, 2021; Wohlfahrt *et al.*, 2018).

Differences in COS and CO _2_ responses to changes in *T _leaf_* and VPD were observed under constant light conditions, but their causes remain unclear ( Kooijmans *et al.*, 2019; Stimler *et al.*, 2010; Sun *et al.*, 2018). Kooijmans *et al.* (2019) measured that LRU decreases with increasing temperatures (from 13 to 23°C) or increasing VPD (from 0.5 to 2.5 kPa) at the boreal forest site Hyytiälä in Finland, while CO _2_ uptake remained relatively stable. Understanding these variations is imperative for utilizing COS measurements to infer information about GPP.

To address these open questions, we investigate three potential explanations for the different responses of COS and CO _2_ uptake to increasing *T _leaf_* and VPD under high-light conditions. First, the pronounced reduction in COS leaf uptake compared to CO _2_ uptake at higher *T _leaf_* might be due to a potential COS compensation point ( *Γ _COS_*) at higher *T _leaf_* (Hypothesis 1), which occurs when uptake and production are equal. If the atmospheric mole fraction is higher than *Γ _COS_*, it generally indicates a predominance of uptake over production. Conversely, below this point, production exceeds uptake, resulting in a net release of COS to the atmosphere.

*Γ _COS_* have been reported in a few vascular plants, algae, crops, and lichen fields ( Belviso *et al.*, 2022; Geng & Mu, 2004; Goldan *et al.*, 1988; Kesselmeier & Merk, 1993; Kuhn & Kesselmeier, 2000; Maseyk *et al.*, 2014). These measured *Γ _COS_* values are lower than typical atmospheric COS mole fractions (≈500 pmol mol ^−1^), which is why net COS uptake is observed. Stimler *et al.* (2010) also observed a *Γ _COS_* of 60.7 pmol mol ^−1^, though statistically indistinguishable from zero, and interpreted it as a possible diffusional feedback under high COS mole fractions. More recently, Gimeno *et al.* (2017) demonstrated temperature-dependent COS emissions in nonvascular bryophytes, suggesting that biochemical processes such as protein degradation could contribute to COS release at elevated *T _leaf_.* However, *Γ _COS_* of vascular plants and its temperature dependence remain poorly constrained. We therefore hypothesize that the observed COS uptake results from the coexistence of uptake and production processes, with *Γ _COS_* exhibiting temperature dependence.

Second, COS leaf uptake might respond more strongly to stomatal closure than CO _2_ uptake (Hypothesis 2). Plants react to variations in VPD to optimize their water use efficiency ( Farquhar & Sharkey, 1982). VPD-induced stomatal closure reduces CO _2_ inflow, but CO _2_ is still consumed within the mesophyll cells. Consequently, the leaf’s internal concentration of CO _2_ is reduced, increasing the CO _2_ gradient between internal and ambient air. This larger gradient could sustain CO _2_ uptake despite stomatal closure.

In contrast, the internal COS concentration is assumed to be much lower than ambient levels due to the higher catalytic efficiency of the CA enzyme ( Protoschill-Krebs *et al.*, 1996; Seibt *et al.*, 2010; Tcherkez *et al.*, 2006). Assuming that the internal COS concentration is (near-)zero, this concentration cannot reduce much further when stomata close, unlike the CO _2_ internal concentration. Consequently, in response to stomatal closure, the COS gradient between internal and ambient air will likely not reduce as much as the CO _2_ gradient, and so the COS gradient will not counteract the stomatal closure as is the case for CO _2_. Thus, decreasing *g _sw_* is expected to reduce COS uptake more than CO _2_ uptake.

Third, the enzyme CA could behave optimally at a lower temperature than the RuBisCO enzyme responsible for CO _2_ fixation ( Cho *et al.*, 2023; Stimler *et al.*, 2010). Consequently, when *T _leaf_* rises above the optimum temperature of CA but is still below that of RuBisCO, COS uptake could decrease while CO _2_ uptake still increases (Hypothesis 3). Enzyme activity typically increases with temperature until reaching an optimum temperature, and the rate and the optimum temperature might differ between enzymes. Optimum temperatures for the CA enzyme in previous models simulating leaf and soil COS uptake ranged from 15°C to 40°C ( Cho *et al.*, 2023; Ogée *et al.*, 2016; Sun *et al.*, 2015), significantly lower than the optimal temperature of 50°C reported for RuBisCO ( Salvucci *et al.*, 2001). Together, these hypotheses consider both stomatal and biochemical mechanisms that could explain the differential COS and CO _2_ responses.

Disentangling these hypotheses using field measurements is challenging due to interrelated and simultaneous variations of environmental conditions (e.g. *T _leaf_*, VPD, and *g _sw_*). Laboratory experiments offer the advantage of observing COS and CO _2_ uptake changes at a leaf level under controlled conditions while independently varying environmental factors. To complement the experimental analysis and mechanistically interpret the observed gas-exchange responses, we developed a coupled CO _2_–COS– H _2_O conductance model based on a leaf conductance model previously established for GPP tracers such as the ∆ _17_O and ∆ _47_ isotopic composition of CO _2_ ( Adnew *et al.*, 2020; Adnew *et al.*, 2021; Adnew *et al.*, 2023). This model serves as a diagnostic tool that enables joint optimization of gas-exchange parameters through the shared stomatal pathway for the three gases and allows inference of internal variables, such as intercellular and chloroplast COS mole fractions, that are not directly measurable.

Finally, to validate the three hypotheses, we aim to investigate the existence of COS compensation points and responses of COS and CO _2_ leaf uptake to varying *g _sw_* and *T _leaf_.* We will present measurement results from leaf gas exchange experiments with sunflowers under controlled environmental conditions. We will interpret the experimental results using the coupled leaf conductance model optimized using the experiments’ dataset.

## 2. Materials and methods

### 2.1 Leaf gas-exchange measurements

***2.1.1 Leaf cuvette system.*** We measured deposition velocities of COS and CO _2_ ( *V _COS_* and *V _CO2_*), i.e. fluxes normalized by mole fractions in air, using a leaf cuvette system. These experiments were conducted using sunflower plants ( *Helianthus Annuus* L. cv. ‘Sunsation’), which are C _3_ photosynthesis type plants cultivated in a local plant nursery (Evanthia, Maasdijk, Netherlands). Plants were cultivated in a greenhouse under a day/night temperature regime of 18–21°C during the day and 15–18°C at night, with ventilation applied above 20°C. Germination occurred at 21–24°C. The species *Helianthus annuus* was originally described by Linnaeus ( *Species Plantarum*, Vol. 2, p.904, 1753). Each sunflower plant was used for a maximum of 5 daytime hours to minimize physiological stress caused by prolonged exposure to experimental conditions. Gas exchange measurements were scheduled to align with the plants’ photosynthetic rhythm and were conducted during the local daytime window between 10:00 and 17:00 (local time, the Netherlands).

*T _leaf_* was monitored within the leaf cuvette using a thermocouple touching the abaxial side of the leaf. A synthetic air mixture, composed of 79% nitrogen and 21% oxygen, was mixed and controlled using mass flow controllers (Bronkhorst, Veenendaal, the Netherlands) and humidified using a dew point generator (LI-610, Li-Cor). CO _2_ and COS were supplied from controlled gas cylinders and then added to the air-stream entering the LI-6800 system. CO _2_ mole fractions in the leaf cuvette were kept around 445.1 *±* 3.5 μmol mol ^−1^. COS was introduced from a gas mixture with synthetic air with a COS mole fraction of around 700 nmol mol ^−1^, regulated by a mass flow controller (Analyt-MTC, Müllheim, Germany). Except for the first experiment to check *Γ _COS_*, COS mole fraction levels were intentionally elevated (in the range of 900 to 1100 pmol mol ^−1^), relative to typically atmospheric mole fractions (approximately 500 pmol mol ^−1^) to improve the detectability of COS uptake. Air in the leaf cuvette was mixed by a fan, operated at approximately 10,000 rpm, and the boundary conductance near the leaf surface was maintained at about 2.44 mol m ^−2^ s ^−1^.

To control *g _sw_*, adjustments were made to the VPD. Initial *g _sw_* (mol m ^−2^ s ^−1^) values were estimated by measuring the total conductance of H _2_O ( *g _tw_* (mol m ^−2^ s ^−1^)) and applying a corresponding boundary conductance of H _2_O ( *g _bw_* (mol m ^−2^ s ^−1^)) according to the LI-6800 manual. We assumed equal distribution of stomata between the upper to lower side of the leaf and therefore applied a stomatal ratio of K = 0.5.

COS and CO _2_ mole fractions of the inflowing and outflowing air were measured using a quantum cascade laser spectrometer (QCLS) (Aerodyne Research Inc., Billerica, MA, USA). The air subjected to QCLS analysis was initially passed through a magnesium perchlorate (Mg (ClO _4_) _2_) drying trap to eliminate water vapor. For this purpose, magnesium perchlorate hexahydrate (99% purity; Sigma-Aldrich, Product Code: 309303-100G, Linear Formula: Mg (ClO _4_) _2_·6H _2_O) was used. The water vapor scrubber tubes employed were 50 cc stainless steel tubes with 0.25-inch tube fittings. Measurements of air from cylinders with known COS and CO _2_ mole fractions were performed for hourly calibration.

Once a leaf reached steady-state under the given conditions, data were recorded. The LI-6800 measured every second and was set to log data automatically every 3 minutes. Each log consisted of an average taken over 15 measurements within the preceding 15 seconds. The QCLS was set to automatically record data every second, totaling 150 measurements over each 150-second period. Then, the median over this 150-second interval was taken as the “data point”. Each QCLS interval had a corresponding LI-6800 log.

The air was passed through the analyzer at an approximate flow rate (AF (mol s ^−1^)) of 0.35 mmol s ^−1^. Subsequently, leaf assimilation rates ( *F _gas_*) of COS ( *F _COS_* (pmol m ^−2^ s ^−1^)) and CO _2_ ( *F _CO2_* (μmol m ^−2^ s ^−1^)) were calculated by:

$$F_{gas}=\frac{AF}{S}({\left[gas\right]}_{in}-{\left[gas\right]}_{out}).$$ (1)

Here, the [gas] _in_ and [gas] _out_ represent the mole fractions of COS (pmol mol ^−1^) and CO _2_ (μmol mol ^−1^) in the air entering and exiting the chamber, which are measured by the QCLS on a dry-air basis. The leaf area *S* (m ^2^) was 9 cm ^2^. Although transpiration slightly increases the outlet air flow due to the addition of water vapor ( von Caemmerer & Farquhar, 1981, Appendix 2), the resulting increase in total molar flow (<3% under [H _2_O] _out_ = 15–30 mmol mol ^–1^) was neglected in Equation 1 for simplicity. This small effect was not considered in the flux calculations and was consistently omitted throughout the model analysis. The CO _2_ leaf assimilation rates obtained from the QCLS measurements closely matched those from the LI-6800 ( *R* = 0.96, mean difference = 0.18 μmol m ^−2^ s ^−1^), confirming the accuracy of the CO _2_ flux measurements.

Hourly calibration corrected potential QCLS drift, and the data points were excluded when the dew point temperature approached or exceeded the air temperature in any experiment.

**Figure 1.**
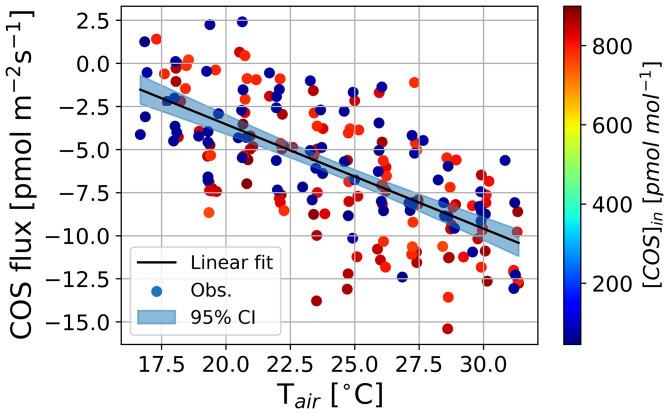
Measurements of COS flux at different air temperatures in the empty chamber. Dots represent measurement data points and their colors indicate injected COS mole fractions in a chamber, black lines are linear fit equations, and filled areas indicate 95% confidential interval.

To account for COS emissions at higher temperatures from chamber material ( Maseyk *et al.*, 2014), we conducted empty chamber experiments using inflowing COS mole fractions of 750 – 900 pmol mol ^−1^, which were adequate for evaluating chamber emissions, across a similar range of temperatures with experimental conditions. Additional experiments with low mole fraction (40 – 75 pmol mol ^−1^) were conducted to evaluate potential emission increases originating from large concentration gradients between sampling air and chamber materials. Figure 1 shows a slight increase in COS emissions with rising temperature, with no significant effect from inflowing COS mole fractions. Measurements were conducted under 40% relative humidity (RH), to avoid potential condensation or other artifacts during the empty chamber experiment. Leaf COS flux measurements were corrected using a linear fit of emissions against temperature in the empty chamber ( *R* ^2^ = 0.45) with chamber emissions averaging 6.7% of observed leaf COS flux.

***2.1.2 Experiments.*** Table 1 lists the environmental factors that were used in the three experiments. We manipulated inflowing COS mole fractions, humidity, and air temperatures while keeping other environmental conditions constant, as specified in each experimental design. All environmental changes were introduced gradually to allow the leaf sufficient time to adapt to chamber conditions. For instance, we applied humidity (to achieve a desired *g _sw_*) and temperature changes of approximately 0.8 mol m ^−2^ s ^−1^ and 10°C, respectively, over a period of two hours. This gradual adjustment ensured that measurements reflect steady-state conditions and minimize transient effects from rapid environmental changes. To exclude the effects of light on *V _COS_* and *V _CO _2__*, high light intensity similar to growth conditions (600 μmol m ^−2^ s ^−1^) was maintained throughout all experiments.

**Table 1.** Environmental conditions during the three experiments. The range of controlled and constant variables for each experiment is expressed as minimum ~ maximum and mean ± standard deviation, respectively. Experiment 1 (Sunflower 1) aims to detect COS compensation points at two temperatures. Experiments 2 (Sunflower 2, 3, and 4) and 3 (Sunflower 2 and 4) are designed to investigate responses of V _COS_ and V _CO2_ to stomatal conductance (g _s_) and leaf temperature ( *T _leaf_*), respectively, with *T _leaf_* acting as a primary driver of mesophyll conductance (g _m_). We controlled a designated variable in each experiment while other variables including light intensity, air flow rate, and mixing fan speed were kept constant.

Experiment (Control)	Experiment 1 ([COS] _in_)		Experiment 2 ( *g*_*sw*_)			Experiment 3 ( *T*_*leaf*_)	
Sunflower number (-)	1	1	2	3	4	2	3
*g _sw_* (mol m ^−2^s ^−1^)	0.8 ± 0.0	0.9 ±0.1	0.4 ~ 1.1	0.3 ~ 1.0	0.3 ~ 0.6	0.6 ± 0.1	0.6 ± 0.0
*VPD* (kPa)	0.5 ± 0.0	0.5 ± 0.0	0.6 ± 0.1	0.7 ± 0.2	0.9 ± 0.3	0.7 ± 0.2	0.9 ± 0.3
*T _leaf_* (°C)	19.8 ± 0.0	25.0 ±0.3	25.1 ± 0.0	26.4 ± 0.1	24.8 ± 0.3	19.1 ~ 30.9	19.0 ~ 29.8
[COS] _in_ (pmol m ^−2^s ^−1^)	94.11 ~ 589.05	104.50 ~ 706.96	1096.4 ± 12.6	1181.9 ± 11.1	914.4 ± 29.6	1087.7 ± 41.1	926.8 ± 14.0
[CO _2_] _in_ (μmol m ^−2^s ^−1^)	446.5 ± 0.1	449.1 ± 0.3	441.0 ± 0.4	446.2 ± 1.5	447.5 ± 1.7	439.9 ± 3.1	447.3 ± 1.4

The first experiment (Experiment 1) aimed to detect COS compensation points and their temperature dependence. To determine the existence of *Γ _COS_* and its temperature response, we measured *F _COS_* at four distinct inflow COS mole fractions ranging from 94 to 589 pmol mol ^−1^ at two different leaf temperatures (19.8°C and 25.0°C). As in Gimeno *et al.* (2017), the values of the *Γ _COS_* were subsequently calculated for each temperature by extrapolating the COS mole fraction at which *F _COS_* reaches zero using a linear regression.

The second experiment (Experiment 2) aimed to compare responses of COS and CO _2_ uptake to stomatal closure. We examined the responses of *V _COS_* and *V _CO _2__* to *g _sw_* while keeping other environmental factors constant, including air temperature (approximately 25°C). Thus, changes in *V _COS_* and *V _CO2_* predominantly reflect responses to changes in *g _sw_.* The modulation of *g _sw_* was accomplished by adjusting humidity within the leaf cuvette – hence modifying VPD – using the humidifier.

The last experiment (Experiment 3) aimed to compare responses of COS and CO _2_ uptake to changing temperatures. We observed *V _COS_* and *V _CO _2__* while controlling the air temperature ( *T _air_*) within the leaf cuvette and maintaining other variables constant, including *g _sw_.* The desired *g _sw_* value was sustained by manipulating chamber humidity and thus VPD when necessary. The resulting leaf temperatures varied between 19.0 and 30.9°C.

Regarding a repetition of experiments, Experiment 1 was executed once with a single sunflower plant (Sunflower 1), while Experiments 2 and 3 were replicated with three different sunflower plants over three days (Sunflower 2, 3, and 4). During the measurement of COS, CO _2_, and H _2_O mole fractions in Experiment 3, the complete temperature range could not be covered consistently across all three repetitions due to fluctuating *g _sw_* conditions. Due to this limitation, data from Sunflower 3 were omitted from the analysis of Experiment 3. Overall, we collected 15 data points from Experiment 1 and 48 data points from Experiments 2 and 3.

The measured *V _COS_* and *V _CO _2__* were used to calculate the LRU (-), which was used to characterize differing responses of COS and CO _2_ from Experiments 2 and 3 ( Sandoval-Soto et al., 2005; Campbell et al., 2008):

$$LRU=\frac{V_{COS}}{V_{CO2}}=\frac{A_{COS}}{{\left[COS\right]}_{out}}\frac{{\left[CO_{2}\right]}_{out}}{A_{CO2}},$$ (2)

where *A _COS_* (pmol m ^−2^ s ^−1^) and *A _CO2_* (μmol m ^−2^ s ^−1^) are assimilation rates of COS and CO _2_, respectively. These rates were normalized by outflowing concentrations [COS] _out_ and [CO _2_] _out_, which are assumed to be the concentrations the plants are exposed to due to the well-mixed conditions (see Section 2.2.1).

### 2.2 Conductance model

***2.2.1 General concept.*** Since the observations were somewhat limited in exploring internal leaf processes, we developed a conductance model and applied an optimization method using 48 data points from Experiments 2 and 3. Experiment 1 (15 data points) was used to provide initial information of *Γ _COS_* and its temperature dependence to the model (Section 3.2.2). This modeling framework was designed to mechanistically link experimental observations to internal gas-exchange processes.

Our leaf conductance model simulates the concurrent exchange of COS, CO _2_, and H _2_O in a plant leaf with the conditions of the laboratory experiments. Since these gases share the same stomatal pathway, their simultaneous modeling stomatal pathway, their simultaneous modeling helps us understand the mechanisms of leaf conductance. The model calculates mole fractions on a wet-air basis, while the measurements are reported on a dry-air basis. Accordingly, conversions between dry- and wet-air mole fractions were applied when comparing modeled and observed concentrations (see Section 2.1.1). Additionally, the model assumes that gas exchange reaches an equilibrium that we tried to attain in the conducted experiments. All model variables that remain constant in the model are listed in Table 2. Variables that are excluded from Table 2 are targets for optimization as explained in Section 2.3.

**Table 2.** Model constants used in the model. Variables in all gases were used for all models, while those specific to COS, CO _2_, and H _2_O were applied to their respective model. The molar volume ( *V _m_*) is at standard temperature (298 K) and pressure (100 kPa).

Gas	Symbol	Description	Unit	Value	*Q*_**10**_
All	*S*	Leaf area	cm ^2^	9.0	
	*R*	Universal molar gas constant	J K ^−1^ mol ^−1^	8.314	
	*V _m_*	Molar volume	m ^3^ mol ^−1^	0.0248	
	*V _cν_*	Cuvette volume	cm ^3^	109 ^a^	
COS	∆ *H _a,CA_*	Activation energy of CA	kJ mol ^−1^	40 ^b^	
	∆ *H _eq,CA_*	Enthalpy change of CA	kJ mol ^−1^	100 ^b^	
CO _2_	*τ*_298 *K*_	Specificity factor between CO _2_ and O _2_ at 298 K	-	2600 ^c^	0.57
	*K*_*c,* 298 *K*_	Michaelis-Menten constant for carboxylation at 298 K	Pa	30 ^c^	2.1
	*K*_*o,* 298 *K*_	Michaelis-Menten constant for oxygenation at 298 K	Pa	30000 ^c^	1.2
	*R _d_*	Dark respiration at 298 K	μmol m ^−2^ s ^−1^	3.0 ^c^	2.0
	*p*[ *O*_2_] *_i_*	Partial pressure of internal O _2_	Pa	20900 ^c^	
H _2_O	*g _mw_*	Mesophyll conductance for H _2_O	mol m ^−2^ s ^−1^	10	

^a^ LI-COR-6800 documents.

^b^ Cho *et al.* (2023).

^c^ Collatz *et al.* (1991).

**Figure 2.**
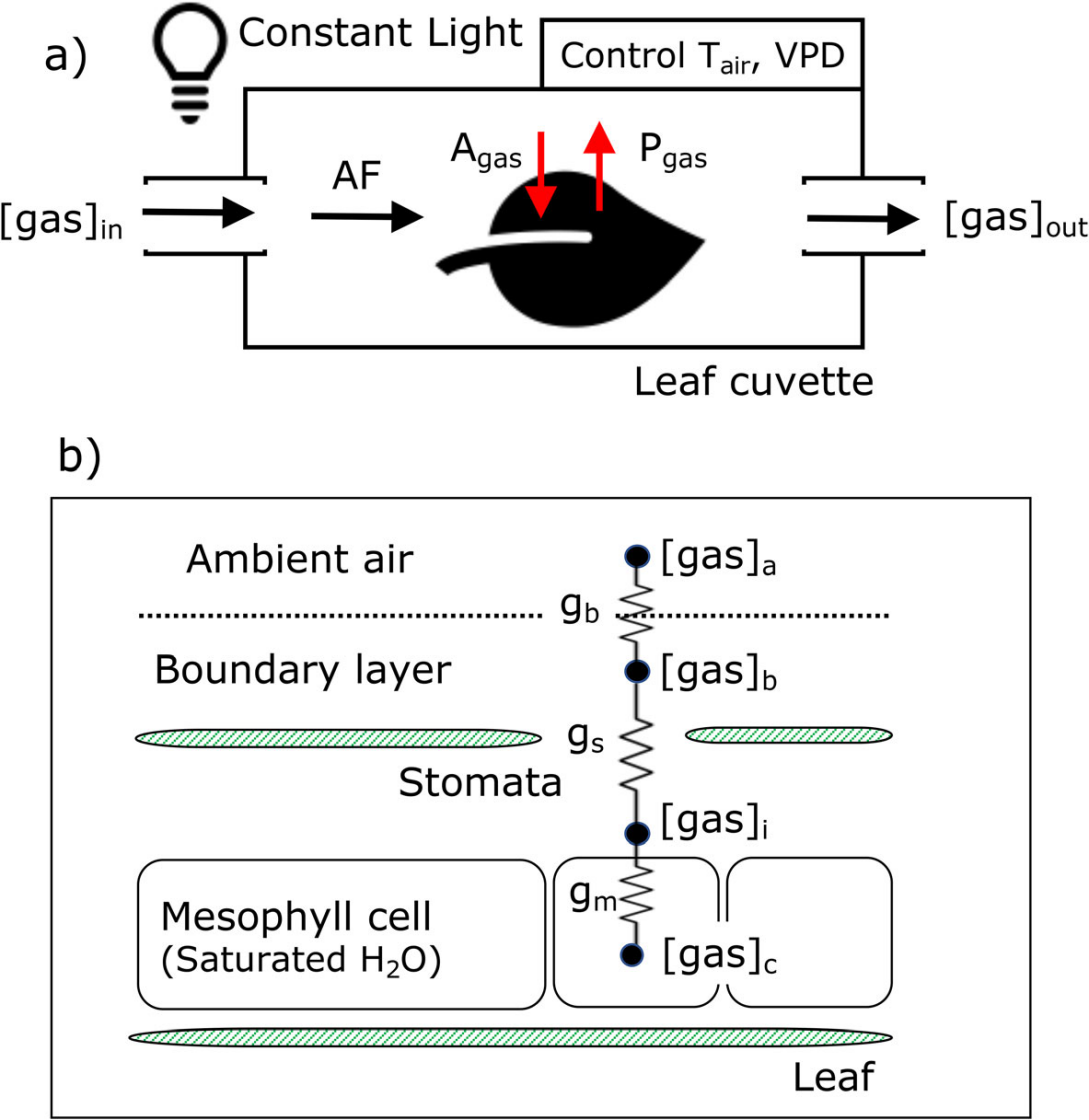
Schemes of leaf cuvette experiments (a) and diffusion pathways of COS, CO _2_, and H _2_O into and out of a leaf in the conductance model (b). A _gas_ and P _gas_ indicate the assimilation rate and production rate of each gas. g _b_, g _s_, and g _m_ represent boundary, stomatal, and mesophyll conductance, respectively.

Figure 2a schematically illustrates the methodology for the gas exchange experiments. As described in Section 2.1.2, environmental conditions, including [COS] _in_, *T _air_*, and VPD, were manipulated to test the hypotheses while keeping other variables, such as light levels, constant. The cuvette received controlled mole fractions of the three gases via the airflow. Figure 2b depicts the exchange pathways of COS, CO _2_, and H _2_O within the sunflower leaf, which form the basis for the conductance model. We assumed that COS, CO _2_, and H _2_O production or consumption within the plant were governed by three conductances: boundary layer conductance ( *g _b_* (mol m ^−2^ s ^−1^)), stomatal conductance ( *g _s_* (mol m ^−2^ s ^−1^)), and mesophyll conductance ( *g _m_* (mol m ^−2^ s ^−1^)) limited by physical or biochemical processes

With a designated set of conductance values, the model simulates the mole fractions of the three gases in each layer: ambient air ([ *gas*] *_a_*), boundary layer ([ *gas*] *_b_*), and inter-cellular airspace ([ *gas*] *_i_*)), and mesophyll level ([ *gas*] *_c_*). The mole fractions of COS, CO _2_, and H _2_O are represented in units of pmol mol ^−1^, μmol mol ^−1^, and mmol mol ^−1^, respectively.

The following rate equations describe the gas exchanges tendencies, assuming they achieve a steady state:

$$\frac{d{\left[gas\right]}_{a}}{dt}\left(\frac{V_{cv}}{V_{m}S}\right)=-g_{b}({\left[gas\right]}_{a}-{\left[gas\right]}_{b})+\frac{AF}{S}({\left[gas\right]}_{in}-{\left[gas\right]}_{a})=0,$$ (3)

$$\frac{d{\left[gas\right]}_{b}}{dt}\left(\frac{V_{cv}}{V_{m}S}\right)=g_{b}({\left[gas\right]}_{a}-{\left[gas\right]}_{b})-g_{S}({\left[gas\right]}_{b}-{\left[gas\right]}_{i})=0,$$ (4)

$$\frac{d{\left[gas\right]}_{i}}{dt}\left(\frac{V_{cv}}{V_{m}S}\right)=g_{S}({\left[gas\right]}_{b}-{\left[gas\right]}_{i})-g_{m}({\left[gas\right]}_{i}-{\left[gas\right]}_{c})=0.$$ (5)

Here, *V _m_* (m ^3^ mol ^−1^) is the molar volume, and *V _cv_* (m ^3^) is the effective air volume representing the gas-exchange region near the leaf surface. Under steady-state conditions, the first term on the left-hand side of Equation 3, Equation 4, and Equation 5, which represents the rate of gas accumulation, becomes negligible. To account for the different properties of COS and CO _2_ to H _2_O, the stomatal conductances ( *g _s,COS_* and *g*_*s,CO*_2__) and the boundary layer conductances ( *g _b,COS_* and *g*_*b,CO*_2__) are scaled proportionally to the conductance of water vapor ( *g _sw_* and *g _bw_*) ( *g*_*s,CO*_2__ = *g _sw_*/1.6, *g*_*b,CO*_2__ = *g _bw_*/1.4, *g _s,COS_* = *g _sw_*/1.94, *g _b,COS_* = *g _bw_*/1.56) ( Bonan, 2008; Seibt *et al.*, 2010; Stimler *et al.*, 2010).

For the COS and CO _2_ exchange at the stomatal level, a ternary system with H _2_O and air should be considered, because the transpiration ( *F*_H _2_O_ (mol m ^−2^ s ^−1^)) is significantly larger than COS and CO _2_ assimilation ( Jarman, 1974; von Caemmerer & Farquhar, 1981). Thus, we added the ternary term in Equation 4 and Equation 5 for the COS and CO _2_ models:

$$\frac{d{\left[gas\right]}_{b}}{dt}\left(\frac{V_{cv}}{V_{m}S}\right)=g_{b}({\left[gas\right]}_{a}-{\left[gas\right]}_{b})-g_{S}({\left[gas\right]}_{b}-{\left[gas\right]}_{i})+\frac{F_{H_{2}O}({\left[gas\right]}_{b}+{\left[gas\right]}_{i})}{2}=0,$$ (6)

$$\frac{d{\left[gas\right]}_{i}}{dt}\left(\frac{V_{cv}}{V_{m}S}\right)=g_{s}({\left[gas\right]}_{b}-{\left[gas\right]}_{i})-\frac{F_{H_{2}O}({\left[gas\right]}_{b}+{\left[gas\right]}_{i})}{2}-g_{m}({\left[gas\right]}_{i}-{\left[gas\right]}_{c})=0.$$ (7)

Although accounting for the H _2_O ternary system is important, its influence on the calculated mole fractions was found to be negligible. The smaller ternary effects within the leaf boundary layer were therefore not included, as they scale with *E*/ *g _bw_* ( von Caemmerer & Farquhar, 1981, Equation B10) and are expected to be negligible under our experimental conditions (large *g _bw_* and small *E*). Note that the mole fractions calculated by the model are expressed on a wet-air basis, whereas the observed mole fractions are reported on a dry-air basis. The moisture correction applied to model outputs is described at the end of this section.

To test Hypothesis 2, we analyzed how the intercellular mole fraction [ *gas*] *_i_* influences the net flux. The flux was computedusing Equation 3, Equation 6, and Equation 7 to determine [ *gas*] *_i_*, and then applied to the flux-gradient relationship:

$$F_{gas}=g_{b∼s}({\left[gas\right]}_{a}-{\left[gas\right]}_{i}).$$ (8)

Here, *g _b∼s_* denotes the conductance from the leaf boundary layer to the stomata. The deposition velocity ( *V _gas_* (mol m ^−2^ s ^−1^)) can be calculated as follows:

$$V_{gas}=g_{b∼s}(1-\frac{{\left[gas\right]}_{i}}{{\left[gas\right]}_{a}}).$$ (9)

The term (1-[gas] _i_ [gas] _a_^−1^) represents the effect of changes between internal and ambient mole fractions on the gas conductance and deposition velocity, termed Ambient Fraction Remaining (AFR). This concept is utilized in Section 3.2.3 for interpreting Hypothesis 2. Strictly speaking, the mass flow difference between the inlet and outlet and the ternary effect should be considered in Equation 8 and Equation 9. However, these effects are expected to be minor under our experimental conditions (see above), and Equation 8 and Equation 9 were used only to examine the conceptual relationship between [gas] _i_ and flux. Therefore, for simplicity, these correction terms were omitted. Note that this omission does not affect the interpretation of AFR in Section 3.2.3.

This set of coupled rate equations can be solved analytically, resulting in mole fractions of the three gases in each layer. Conductance at the mesophyll level ( *g _m_*) and [ *gas*] *_c_* were calculated using a different model for each gas. Descriptions of the individual biophysical processes at the mesophyll level for each gas are provided in subsequent sections.

From experiments, we measured [ *gas*] *_out_*, while the leaf conductance model calculates [ *gas*] *_a_.* Because the chamber air was well mixed, the modeled [ *gas*] *_out,est_* is considered equivalent to [ *gas*] *_a_* for model-observation comparison. Thus, [ *gas*] *_out,est_* is used for [ *gas*] *_a_* to interpret experimental data.

In the model, the estimated mole fractions for COS and CO _2_ are based on wet air, whereas the QCLS measures dry-air mole fractions. Therefore, to compare model results with observations, the modeled wet-air mole fractions were converted to their dry-air equivalents using the observed outgoing H _2_O mole fractions ([H _2_O] _out,obs_ (mmol mol ^−1^)):

$${\left[gas\right]}_{out,est}={\left[gas\right]}_{a}{\left(1-\frac{{\left[H_{2}O\right]}_{out,obs}}{1000}\right)}^{-1}.$$ (10)

For [COS] _in,obs_, which was measured after moisture removal, the same correction ( Equation 10) was applied using [H _2_O] _in,obs_ instead of [H _2_O] _out,obs_ to convert from the dry-air to the wet-air basis before use in the conductance model.

Finally, net fluxes of the three gases were calculated using the same methodology as employed for the observations ( Equation 1).

***2.2.2 Water vapor model.*** In our experiments, we observed water evaporation from leaves. We aimed to model this evaporation at the mesophyll level and its subsequent transport out of the leaves, governed by *g _sw_.* The unit of H _2_O mole fractions in all layers is mmol mol ^−1^, and the unit of the flux is mmol m ^−2^ s ^−1^.

To represent the internal water vapor conditions, [H _2_O] _c_ was calculated assuming that water vapor within the mesophyll intercellular airspace is saturated. However, we allowed for relative humidity within the intercellular airspace (RH _i_ (%)) to be less than 100%, based on previous studies ( Cernusak *et al.*, 2018; Wong *et al.*, 2022). To achieve this, we introduced a mesophyll conductance for water vapor ( *g _mw_*) and set it to an arbitrary value of 10 mol m ^−2^ s ^−1^, approximately ten times the largest observed value of *g _sw_.* This parameter was included to test the sensitivity of the model to possible non-saturation within the intercellular airspace.

The expression for [H _2_O] _c_ relies on *T _leaf_* (°C) and air pressure ( *P _air_* (Pa)):

$${\left[H_{2}O\right]}_{c}=1000\;\frac{P_{H_{2}O}}{P_{air}},\;\text{with}$$ (11)

$$P_{H_{2}O}=613.5\;\text{exp}\;(\frac{17.502\;T_{leaf}}{T_{leaf}+240.97}).$$ (12)

Using these assumptions, we calculated the water vapor mole fractions in the atmosphere ([H _2_O] _a_), boundary layer ([H _2_O] _b_), and interior ([H _2_O] _i_) by solving Equation 3, Equation 4, and Equation 5. Finally, we estimated the RH _i_ (%) from the calculated [H _2_O] _c_ and [H _2_O] _i_:

$$RH_{i}=100\;\frac{{\left[H_{2}O\right]}_{i}}{{\left[H_{2}O\right]}_{c}}.$$ (13)

***2.2.3 CO***_***2***_ ***model.*** We aimed to simulate the gross CO _2_ flux ( *F _CO _2__*, μmol m ^−2^ s ^−1^) and mole fractions of CO _2_ in each layer (μmol mol ^−1^) using the model proposed by Farquhar *et al.* (1980). In this model, the gross CO _2_ uptake rate is calculated as the minimum value among assimilation limited by enzymes ( *A _m_*), light ( *A _E_*), and sucrose synthesis ( *A _S_*), minus the dark respiration ( *R _d_*) :

$$F_{CO2}\;\approx \;\min(A_{m},A_{E},A_{S})-R_{d}.$$ (14)

Our experiments were conducted under a constant light intensity (PAR = 600 μmol m ^−2^ s ^−1^). We assumed that the *A _E_* and *A _S_* pathways were saturated, as light saturation points previously were obtained above 500 μmol m ^−2^ s ^−1^ ( Lobo *et al.*, 2013). Consequently, we calculated *g*_*m,CO*_2__ ([CO _2_] _i_ − [CO _2_] _c_) in Equation 7 with *A _m_ −R _d_.* *R _d_* was estimated by Collatz *et al.* (1991).

*A _m_* is calculated by multiplying the maximum rate of RuBisCO carboxylation ( *V _max,Rub_*) with the partial pressure changes from RuBP partitioning between the CO _2_ carboxylation and O _2_ oxygenation reactions:

$$A_{m}=\frac{V_{max,Rub}\;f_{rub}(T_{leaf})(p{\left[CO_{2}\right]}_{i}-\Gamma_{CO_{2}}^{*})}{p{\left[CO_{2}\right]}_{i}+K_{C}(1+\frac{p{\left[O_{2}\right]}_{i}}{K_{o}})},$$ (15)

where *p* [CO _2_] _i_ (Pa) and *p* [O _2_] _i_ (Pa) are the partial pressures of CO _2_ and O _2_ in the inter-cellular air space. *K _c_* (Pa) and *K _o_* (Pa) are Michaelis-Menten constants for CO _2_ and O _2_, respectively. *Γ* ^∗^ *_CO _2__* (Pa) stands for the CO _2_ compensation point independent of dark respiration.

Strictly speaking, RuBisCO carboxylation occurs within the chloroplast stroma and should depend on the chloroplast CO _2_ partial pressure ( *p* [CO _2_] _c_), rather than *p* [CO _2_] _i_. However, our model does not explicitly include mesophyll conductance ( *g*_*m,CO*_2__), and thus *p* [CO _2_] _c_ cannot be directly resolved. Accordingly, *p*[CO _2_] _i_ was used as a proxy for *p* [CO _2_] _c_, following the classical formulation of Farquhar *et al.* (1980).

The estimation of *Γ* ^∗^ *_CO2_* is based on the partial pressure of oxygen linked to the side reaction of RuBisCO:

$$R_{2}=R_{1}\;Q_{10}^{\frac{\left(T_{2}-T_{1}\right)}{10}}.$$ (16)

Here, *T*_2_ corresponds to *T _leaf_*, and *T*_1_ is 25°C for the CO _2_ parameters *K _c_*, *K _o_*, and Γ ^∗^ *_CO _2__.* The temperature function of *V _max,Rub_* (μmol m ^−2^ s ^−1^) at *T _leaf_* (°C) is calculated by ( Badger & Collatz, 1977):

$$f_{Rub}(T_{leaf})=\text{exp}\;(\frac{\left((T_{leaf}+273.15)-T_{ref,Rub}\right)\Delta H_{a,Rub}}{T_{ref,Rub}\;R\;(T_{leaf}+273.15)}),$$ (17)

where ∆ *H _a,Rub_* (J mol ^−1^) is the activation energy of RuBisCO, valued at 60.0 kJ mol ^−1^ ( von Caemmerer & Evans, 2015), and *T _ref,Rub_* (K) is the reference temperature (298 K). *R* is the universal molar gas constant (8.314 J K ^−1^ mol ^−1^).

***2.2.4 COS model.*** We constructed the COS model to estimate the gross COS flux rate ( *F _COS_*, pmol m ^−2^ s ^−1^) and mole fractions of COS in each layer (pmol mol ^−1^). Accurate modeling of COS leaf uptake requires representing mesophyll conductance, which integrates both the diffusional transport of COS and biochemical conversion by CA.

Earlier approaches treated mesophyll diffusion and CA activity as linearly proportional to Rubisco’s *V _max_* ( Berry *et al.*, 2013). However, this simplification was shown to bias COS flux estimates, leading to the introduction of a revised CA-based temperature formulation ( Cho *et al.*, 2023). More recently, Lai *et al.* (2024) emphasized the importance of explicitly accounting for mesophyll diffusion in COS uptake models but also noted the lack of reliable parameterization for COS-specific mesophyll diffusion.

Building on these previous findings, we modeled biochemical conductance of COS in the mesophyll ( *g _m,COS_* (mol m ^−2^ s ^−1^)) using the CA activity–based function proposed by Cho *et al.* (2023), which implicitly represents mesophyll diffusion while minimizing estimation bias through its revised temperature dependence. This function describes the temperature dependence of CA activity using a specified Arrhenius equation and Michaelis-Menten Kinetics ( Cho *et al.*, 2023; Daniel *et al.*, 2010; Ogée *et al.*, 2016; Sun *et al.*, 2015):

$$g_{m,COS}=V_{\text{max},CA}\;f_{CA}(T_{leaf})=V_{\text{max},CA}\;A_{T}\frac{\left(T_{leaf}+273.15)\;\text{exp}[-\frac{\Delta H_{a,CA}}{R(T_{leaf}+273.15)}\right]}{1+\text{exp}\;[-\frac{\Delta H_{eq,CA}}{R}(\frac{1}{T_{leaf}}-\frac{1}{T_{eq,CA}+273.15})]}.$$ (18)

Here, *V _max,CA_* (mol m ^−2^ s ^−1^) is the maximum catalyzation velocity of CA, *∆H _a,CA_* (J mol ^−1^) represents the activation free energy of CA and ∆ *H _eq,CA_* (J mol ^−1^) is the enthalpy change when the enzyme transits from activation to inactivation. *T _eq,CA_* (°C) stands for the optimum temperature at which the concentrations of activated and inactivated enzymes are equal ( Daniel *et al.*, 2010; Ogée *et al.*, 2016; Sun *et al.*, 2015). We adopted the normalization factor *A _T_* as the value of *f _CA_* ( *T _eq,CA_*) ^−1^.

To incorporate temperature-dependent compensation point behavior, applied the *Γ _COS_* into [COS] _c_. We proposed four temperature functions of *Γ _COS_* based on Experiment 1 data. For model S1, *Γ _COS_* was set to zero across all temperatures, assuming no compensation point effects. For model S2, *Γ _COS_* was represented as a linear function:

$$\Gamma_{COS}(T_{leaf})=f_{\Gamma COS}(T_{leaf})=m_{COS}(T_{leaf}-T_{ref}).$$ (19)

where *T _ref_* (°C) is the intercept temperature (16.4°C), determined from the linear regression of two *Γ _COS_* values detected in Experiment 1 (Section 3.1.1). The slope *m*_COS_ (pmol mol ^−1^ K ^−1^) was set to 16.2 pmol mol ^−1^ K ^−1^, also derived from Experiment 1. At low temperatures, where *Γ _COS_* becomes negative, we assumed *Γ _COS_* = 0.

For model S3, *Γ _COS_* was calculated using the Arrhenius function with a observation-based value of 55.0 pmol mol ^−1^ at 293 K from Experiment 1:

$$\Gamma_{COS}(T_{leaf})=55.0\;\text{exp}(\frac{\left((T_{leaf}+273.15)-293\right)\Delta H_{a,\Gamma }}{293\;R\;(T_{leaf}+273.15)}),$$ (20)

and for model S4, *Γ _COS_* was similarly computed using a reference value of 138.7 pmol mol ^−1^ at 298 K:

$$\Gamma_{COS}(T_{leaf})=138.7\;\exp(\frac{\left((T_{leaf}+273.15)-298\right)\text{Δ}H_{a,\Gamma }}{298\;R\;(T_{leaf}+273.15)}),$$ (21)

where *∆H _a,Γ_* (kJ mol ^−1^) represents the activation energy for *Γ _COS_.* By solving for ∆ *H*_*a, *Γ_ using the two derived *Γ _COS_* values, we determined an initial activation energy of 134.3 kJ mol ^−1^.

**Figure 3.**
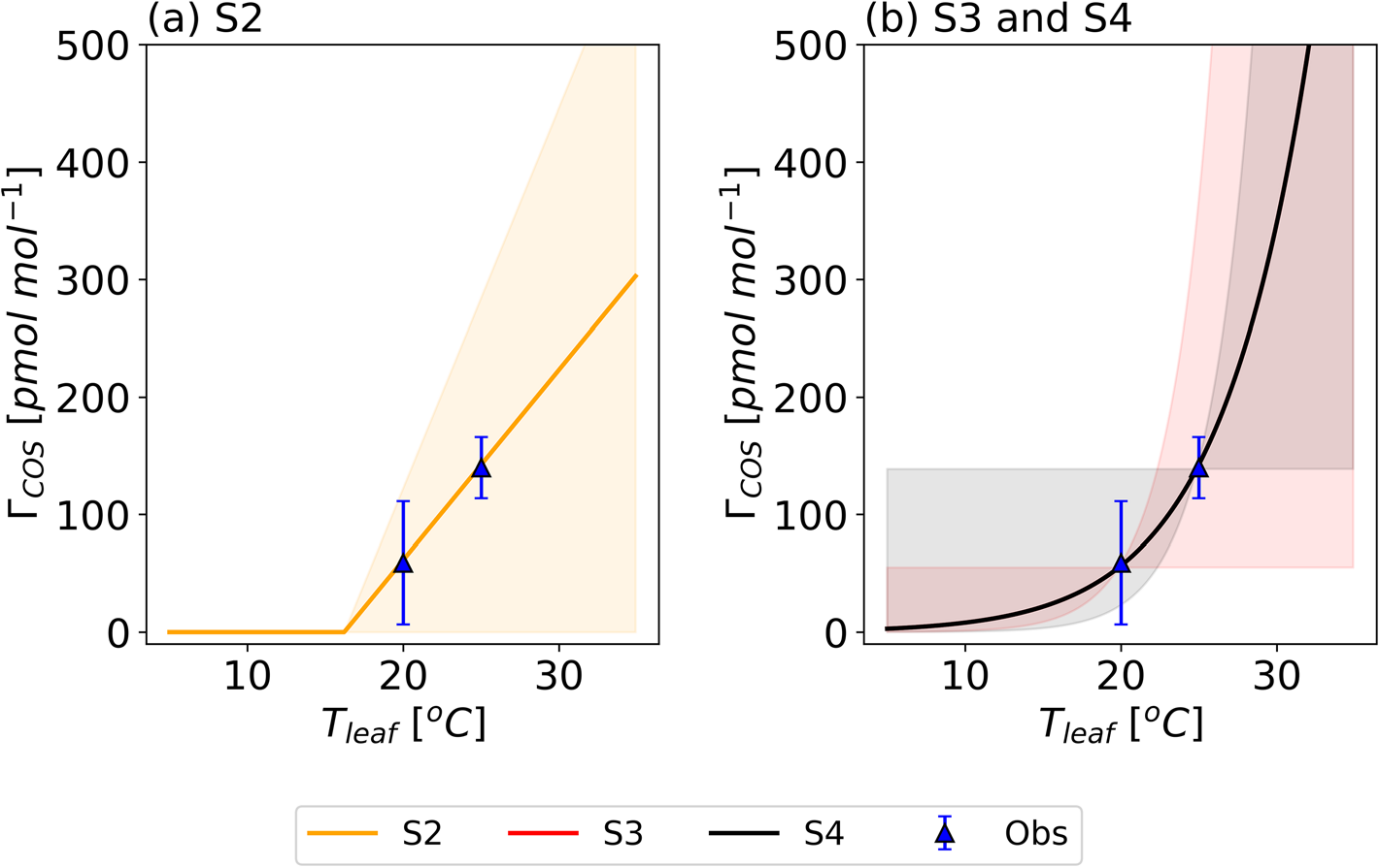
Prior temperature functions of the COS compensation point ( *Γ _COS_*) (lines) with their error ranges (shaded area). Blue triangles show the regression-derived *Γ _COS_* from Experiment 1, included for reference to illustrate the initial parameter setup. The S1 model assumed *Γ _COS_* = 0 pmol mol ^−1^. Model S2 describes *Γ _COS_* as a linear relationship with *T _leaf_* (a), while models S3 and S4 use an Arrhenius equation (b).

Figure 3 shows these four *Γ _COS_* functions (S1–S4) representing the prior temperature-dependent parameterizations tested in the modeling framework with their imposed uncertainties. The figure also shows the experimental *Γ _COS_* estimates from Experiment 1, included solely for reference to illustrate the initial parameter alignment.

### 2.3 Indirectly derived COS compensation point

Because the temperature-dependent behavior of *Γ _COS_* in Experiment 1 was derived from a limited set of measurements under a few temperature levels, additional validation was required to test whether the modeled *Γ _COS_* functions are consistent across broader environmental conditions. To support this validation, we incorporated the indirectly derived *Γ _COS_* from 48 data points in Experiments 2 and 3. By calculating [COS] _c_ using our optimized model parameters in Equation 3, Equation 6, and Equation 7, we were able to indirectly estimate *Γ _COS_* and compare it with the modeled *Γ _COS_.*

First, we calculated [COS] _b_ from the measured COS mole fractions with the rearranged Equation 3:

$${\left[COS\right]}_{b}={\left[COS\right]}_{a}+\frac{AF}{S\;g_{b,COS}}({\left[COS\right]}_{a}-{\left[COS\right]}_{in}),$$ (22)

where [COS] _in_ and [COS] _a_, along with *AF*, *S*, and *g _b,COS_*, were applied from Experiments 2 and 3. Due to thorough mixing within the cuvette, we assumed [COS] _out_ to be equivalent to [COS] _a_ and used it accordingly (refer to Section 2.2). Although transpiration slightly increases the outlet air flow, this effect (<3%) was neglected to retain easy analytical expressions, consistent with the assumptions described in Equation 1. This formulation follows the same assumptions as in Equation 3 regarding ternary effects at the boundary layer, which were found to be negligible under our experimental conditions. Here, [COS] _in_ and [COS] _out_ were converted from a dry air basis to a wet air basis. We then used the derived [COS] _b_ to calculate [COS] _i_ using the rearranged Equation 6:

$${\left[COS\right]}_{i}=(-g_{b,COS}{\left[COS\right]}_{a}+g_{b,COS}{\left[COS\right]}_{b}+g_{s,COS}{\left[COS\right]}_{b}-0.5\;F_{H2O}{\left[COS\right]}_{b}){\left(g_{s,COS}+0.5\;F_{H_{2}O}\right)}^{-1}.$$ (23)

*F*_H _2_O_ was calculated using Equation 1 with the observed [H _2_O] _in_ and the modeled [H _2_O] _a_ obtained by the H _2_O model. We used optimized *g _sw_* value in the the H _2_O model and *g _s,COS_.* The reason for using the model-calculated [H _2_O] _a_ instead of the observed [H _2_O] _out_ is to consider the optimized *g _sw_* in *F*_H _2_O_.

Finally, [COS] _i_ is used in Equation 7 to derive [COS] _c_:

$${\left[COS\right]}_{c}=-\frac{g_{s,COS}}{g_{m,COS}}({\left[COS\right]}_{b}-{\left[COS\right]}_{i})+\frac{F_{H2O}}{g_{m,COS}}({\left[COS\right]}_{b}+{\left[COS\right]}_{i})+{\left[COS\right]}_{i},$$ (24)

where *g _m,COS_* was determined using Equation 18 with optimized parameters *V _max,CA_* and *T _eq,CA_.* These indirectly calculated *Γ _COS_* values vary depending on the chosen temperature-dependent functions of *Γ _COS_*, which influence other parameters. Consequently, the indirectly derived *Γ _COS_* values differ from *Γ _COS_* functions due to their impact on the optimized parameters used in the calculations.

### 2.4 Parameter optimization

***2.4.1 Optimization setting.*** Beginning with the model state parameter ( *x*), we calculated [ *gas*] *_out,est_* and compared these to the observed values [ *gas*] *_out,obs_* on a dry-air basis. We aimed to minimize the differences between our model and the observations during each optimization step. To accomplish this, we defined a cost function ( *J _tot_*) consisting of a background term ( *J _bg_*) and three observational terms that account for the differences between model estimations and observations of COS ( *J*_COS_), CO _2_ ( *J*_CO _2__), and H _2_O ( *J*_*H*_2_ *O*_):

$$J_{tot}=J_{bg}+J_{COS}+J_{CO_{2}}+J_{H_{2}O}=\frac{{\left(x-x_{a}\right)}^{2}}{W_{bg}\sigma^{2}}+\frac{{\left({\left[COS\right]}_{out,est}-{\left[COS\right]}_{out,obs}\right)}^{2}}{W_{COS}{\left(\sigma_{COS}\right)}^{2}}+\frac{{\left({\left[CO_{2}\right]}_{out,est}-{\left[CO_{2}\right]}_{out,obs}\right)}^{2}}{W_{CO_{2}}{\left(\sigma_{CO_{2}}\right)}^{2}}+\frac{{\left({\left[H_{2}O\right]}_{out,est}-{\left[H_{2}O\right]}_{out,obs}\right)}^{2}}{W_{H_{2}O}{\left(\sigma_{H_{2}O}\right)}^{2}}.$$ (25)

The first term of the cost function penalizes deviations of the state *x* from the prior state vector *x _a_.* This penalty depends on *σ*, which represents the prior error in the state vector. This background term is introduced to keep state variables within reasonable boundaries ( Brasseur & Jacob, 2017). The last three terms of the cost function calculate the costs associated with deviations between modeled ([ *gas*] *_out,est_*) and observed mole fractions ([ *gas*] *_out,obs_*) for for COS, CO _2_, and H _2_O, respectively. Each term is normalized by its observational uncertainty to ensure consistent treatment of measurement errors, and each is scaled by optimization weights (W _bg_, W _COS_, W _CO _2__, and W _H _2_O_) to balance the relative influence of each gas. These weights do not modify the observational errors but instead prevent any single gas—particularly CO _2_—from dominating the total cost. The determination of these weights is described in the following section.

The costs related to observations ( *J*_COS_, *J*_CO _2__ , and *J*_H _2_O_) are influenced by observational errors ( *σ*_COS_, *σ*_CO _2__ , and *σ*_H _2_O_), derived from the measured COS, CO _2_, and H _2_O mole fractions. These observational errors were determined as the standard deviation of the measured mole fractions at outflow within the data point interval (150-second). Formally, *σ*_COS_, *σ*_CO _2__, and *σ*_H _2_O_ should also reflect model errors caused by model parameters not included in the state or by processes that were not modeled ( Brasseur & Jacob, 2017). Note that we excluded data from Experiment 1 to validate the optimized temperature function of *Γ _COS_* in Section 3.2.2.

Due to the larger relative error in [H _2_O] _out,obs_ and [COS] _out,obs_ than in [CO _2_] _out,obs_, the optimization procedure tended to focus on minimizing preliminary *J*_CO _2__. To better utilize the non-CO _2_ observations, we introduced optimization weights for each cost component. The observed [CO _2_] _out,obs_ values ranged from 379.4 to 390.0 μmol mol ^−1^, with an average observational error of only 0.2 μmol mol ^−1^ (0.05%). [COS] _out,obs_ values ranged from 748.2 to 1032.4 pmol mol ^−1^, with an average error of 16.7 pmol mol ^−1^ (1.91%). [H _2_O] _out,obs_ values ranged from 15.2 to 32.5 mmol mol ^−1^ with a mean error of 0.3 mmol mol ^−1^ (1.14%).

The targeted state parameters, along with their initial values and errors, are listed in Table 3. We assumed that the parameters related to the temperature response of mesophyll cells are consistent across all experiments, as all sunflowers were cultivated in a similar climate. We constructed state vectors with variables that significantly impact the total cost and better quantify the combined leaf exchanges: *V _max,CA_* , *T _eq,CA_*, and *m*_COS_ for COS, *V _max,Rub_* and ∆ *H _a,Rub_* for CO _2_, and *g _sw_* for all gases.

**Table 3.** State parameters ( *x*) and their prior and posterior values with their errors based on model S2. Posterior errors are calculated from 200 optimizations in a Monte Carlo method ( Section 2.5). The last column denotes the error reduction. Prior and Posterior values for *g _sw_* are averaged values across the dataset from Experiments 2 and 3. Values in “Bounds” column with brackets indicate lower and higher bounds provided to the optimization.

Parameter	Description	Unit	Bounds	Prior ± Error	Posterior ± Error	(b-a)/a ^**1**^ (%)
*V _max,CA_*	Maximum velocity for CA	mol m ^−2^ s ^−1^	[0.01, 0.50]	0.125 ± 0.060	0.217 ± 0.037 ^2^	38
					0.189 ± 0.029 ^3^	52
					0.230 ± 0.042 ^4^	30
*T _eq,CA_*	Optimum temperature for CA	°C	[1, 60]	30.0 ± 15.0	39.7 ± 5.0	66
*m _COS_*	Slope of *Γ _COS_*	pmol mol ^−1^ K ^−1^	[1, 300]	16.2 ± 16.2	22.4 ± 5.3	67
*V _max,Rub_*	Maximum velocity for RuBisCO	*μ*mol m ^−2^ s ^−1^	[1, 200]	90.0 ± 20.0	94.4 ± 0.3 ^2^	98
					87.4 ± 0.5 ^3^	98
					82.9 ± 0.3 ^4^	98
∆ *H _a,Rub_*	Activation energy for RuBisCO	kJ mol ^−1^	[1, 100]	60.0 ± 12.0	54.5 ± 0.9	92
*g _sw_*	Stomatal conductance of H _2_O	mol m ^−2^ s ^−1^	[0, 3]	0.62 ± 0.09	0.64 ± 0.05	43

^1^ ‘a’ is a prior error and ‘b’ is a posterior error. They are used to calculate the error reduction.

^2–4^ The values are obtained for Sunflower 2, 3, and 4, respectively.

Although we selected sunflower leaves at similar development stages, variations in biochemical functioning existed among individual leaves. Therefore, we optimized *V _max,CA_* and *V _max,Rub_* for each individual plant, as these largely determine mesophyll conductance ( Cho *et al.*, 2023; Le Roux *et al.*, 2001; Williams & Flanagan, 1996). The initial *V _max,CA_* was based on the averaged *V _max,CA_* across different phenological stages from two observation sites, Hyytiälä (Finland) and Harvard Forest (United States), as introduced by Cho *et al.* (2023). The standard deviation of *V _max,CA_* was used as the state error. For *V _max,Rub_*, we set the initial value as *V _max,CA_* divided into the scaling factor 1400, derived from laboratory measurements of gas exchange in C _3_ plants ( Berry *et al.*, 2013; Stimler *et al.*, 2010; Stimler *et al.*, 2011).

*T _eq,CA_* is more crucial in determining CA’s temperature function compared to other kinetic variables ( Cho *et al.*, 2023). We adopted the initial value and state error of *T _eq,CA_* directly from Cho *et al.* (2023), ensuring consistency with previous characterizations of CA kinetics.

To further evaluate the temperature dependence of *Γ _COS_* , four temperature functions (S1 to S4) were tested within the conductance model. Each function was optimized and compared against observation-based *Γ _COS_.* The S1 model excludes *Γ _COS_*, while S2 applies a linear function with *m*_COS_ of 16.2 pmol mol ^−1^. Both S3 and S4 use a temperature dependence described by an Arrhenius relationship. All models were initialized with the same prior error for *Γ _COS_*, including the zero-compensation point, as shown in Figure 3.

For the CO _2_ parameters, we optimized ∆ *H _a,Rub_*, as it is an unknown parameter for Sunflowers. We determine the initial value and error of ∆ *H _a,Rub_* using the mean and standard deviation across nine species reported by von Caemmerer and Evans (2015). Other temperature-dependent parameters were adopted from Collatz *et al.* (1991).

The LI-6800 derives *g _sw_* from water vapor exchange with its own uncertainties. Because *g _sw_* similarly affects the exchange of H _2_O, CO _2_, and COS through the same shared stomatal pathway, we optimize *g _sw_* in a coupled framework using flux information from all three gases to ensure internal consistency across species. The novelty of this framework lies in explicitly coupling these fluxes to derive a more physically consistent constraint on *g _sw_*, while also addressing potential uncertainties in *g _sw_* derived from LI-6800 measurements (e.g., stomatal ratio parameter K; Section 2.1.1).

Initial values for *g _sw_* were taken from the LI-6800 measurements during Experiments 2 and 3. We applied a random error of 0.08 mol m ^−2^ s ^−1^, representing the standard deviation of *g _sw_* from Experiment 3, despite efforts to maintain a constant *g _sw_* ( *±* 0.06 mol m ^−2^ s ^−1^). In addition, we considered an extra measurement uncertainty of 0.02 mol m ^−2^ s ^−1^, based on known sources of instrumental bias—such as the leaf temperature underestimation ( Garen *et al*., 2022) and background total leaf conductance to water observed in empty chambers ( Hussain *et al.*, 2024). We also added individual [H _2_O] _out,obs_ errors by normalizing [H _2_O] _out,obs_. The averaged initial *g _sw_* value and its standard deviation (prior error) are 0.61 mol m ^−2^ s ^−1^ and 0.09 mol m ^−2^ s ^−1^, respectively.

Assuming a Gaussian probability density function for the state parameters could lead to nonphysical values in the optimization process. Thus, we minimized *J _tot_* using the Sequential Least SQuares Programming optimizer (SLSQP) from the SciPy Python library. This optimizer enables the specification of lower and upper bounds on the state space and is well-suited for solving nonlinear problems. The parameter bounds are listed in Table 3.

***2.4.2 Weight determination.*** We selected weights to achieve more balanced prior costs across four components in Equation 25. The prior chi-squared value ( *χ ^2^_prior_*) was used to check the cost balance in prior condition. We set seven possible weights, and the corresponding *χ ^2^_prior_* value ranged from 3 to 200, as listed in Table 4. Then, we optimized parameters and calculated cost reduction rates for each part of the cost function. For H _2_O, we found a *χ ^2^_prior_* value of about 3, indicating that the H _2_O model already performs well with the prior parameter settings.

**Table 4.** Candidates for weights in the observational terms ( *W _COS_*, *W _CO _2__*, *W _H _2_O_*) based on the chosen *χ ^2^_prior_* values.

*χ* ^*2*^_*prior*_	*W*_*COS*_	*W*_*CO _2_*_	* W*_*H _2_O*_
3	0.337	238.8	3.24
10	0.101	71.6	0.97
30	0.034	23.9	0.32
50	0.020	14.3	0.19
100	0.010	7.2	0.10
150	0.007	4.8	0.06
200	0.005	3.6	0.05

We finally selected the weight combination that minimized the averaged reduction rates of *J*_COS_ and *J*_CO 2_ and ensured that each *χ* ^2^_poste_ exceeded the value of 0.6 to avoid over-fitting, including *J _bg_.* The selected weights ( *W _COS_* = 0.034, *W _CO _2__* = 7.2, *W _H _2_O_* = 3.24, *W _bg_* = 1.0), correspond to *χ* ^2^_prior_ values of 30, 100, and 3 for COS, CO _2_, and H _2_O, respectively. These weights lead to a more balanced ratio of *χ* ^2^ *_prior_* across the observational parts (COS: CO _2_: H _2_O = 10: 33: 1), compared to the ratio without weights (1: 329: 4). Note that the prior value of *J _bg_* is zero by design.

### 2.5 Error statistics

We evaluated the model performance using *χ* ^2^ values, mean bias errors (MBEs), and root mean square errors (RMSEs). The *χ* ^2^ metric was calculated by dividing the partial cost function by the number of observations or state variables ( Tarantola, 2005). A posterior chi-squared value ( *χ* ^2^_poste_) of 1 indicates a good fit, as the model predictions fall within the error distribution fall within the error distribution of the observations. A *χ* ^2^_poste_ significantly greater than 1 indicates a poor fit, while a value smaller than 1 suggests potential overfitting. MBE indicates bias, with a positive value for overestimation and a negative value for underestimation. RMSE is the quadratic mean of the differences between estimation and observation, with a value of 0 indicating a perfect fit.

Note that the optimization process targets for *χ* ^2^_poste_ to reach 1.0, but we also give the improvement in MBEs and RMSEs for completeness. To assess the posterior error associated with the optimized state, we employed a Monte Carlo method. In this process, we optimized the parameters using 200 distinct ensemble members, introducing random noise within the bounds of the respective state and observational errors ( Bosman & Krol, 2023; Chevallier *et al.*, 2007; Cho *et al.*, 2023).

Furthermore, we conducted 500 perturbed forward simulations to assess the spread in both prior and posterior models. Parameters were randomly sampled from Gaussian distributions of both the prior and posterior. Prior uncertainties were assumed uncorrelated, while for the posterior parameters, we used the correlations that were determined using the Monte Carlo optimization. We quantified the posterior covariance matrix and considered the cross-parameter correlations when sampling the parameters to evaluate the posterior model. Parameters more than three standard deviations from the mean were discarded. These simulations were conducted with designated environmental input variables: pressure = 103,100 Pa, air flow rate ( *AF*) = 0.00035 mol s ^−1^, [CO _2_] _in_ = 400 μmol mol ^−1^, [COS] _in_ = 1000 pmol mol ^−1^, [H _2_O] _in_ = 15 mmol mol ^−1^, *g _bw_* = 2.44 mol m ^−2^ s ^−1^, *g _sw_* = 0.6 mol m ^−2^ s ^−1^, and *T _leaf_* = 25°C. We only varied one variable at the time, to evaluate its impact on the simulation.

## 3. Results

### 3.1 Experiments

**Figure 4.**
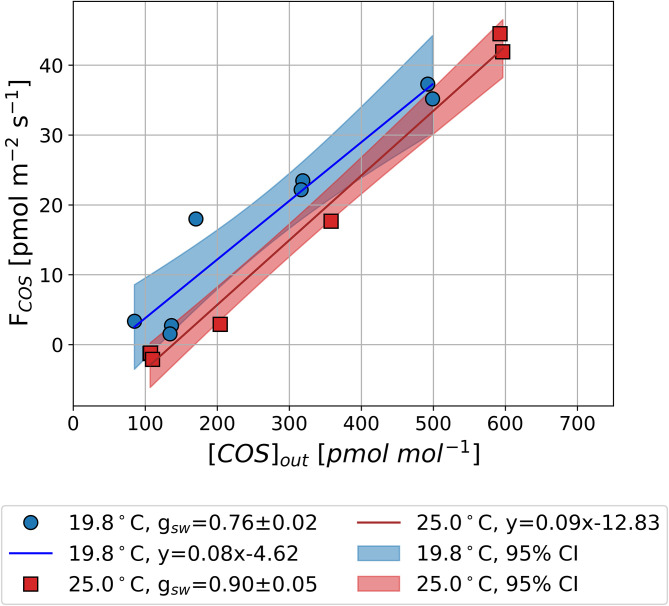
Observed mean COS net flux ( *F _COS_*) along an outflowing COS mole fraction ([COS] _out_) in the light at 25.0°C (rectangle and red areas) and 19.8°C (circle and blue areas) in Experiment 1. Lines and shaded areas represent linear fits with 95% confidence intervals. Positive values in *F _COS_* (y axis) indicate COS uptake, and negative values indicate COS emissions.

***3.1.1 COS Compensation Point (Experiment 1).*** Figure 4 presents the results from Experiment 1 with Sunflower 1, aimed at determining *Γ _COS_* by measuring *F _COS_* while controlling [COS] _out_ and maintaining constant *g _sw_* and *T _leaf_.* Minor fluctuations in *g _sw_* occurred during temperature adjustments, increasing from 0.76 to 0.90 mol m ^−2^ s ^−1^ higher temperatures. *F _COS_* shows a linear increase with increasing [COS] _out_, in agreement with findings from earlier studies ( Gimeno *et al.*, 2017; Kesselmeier & Merk, 1993; Stimler *et al.*, 2010).

We quantified the regression-based *Γ _COS_* using a linear function at two temperatures: *Γ _COS_* was 55.0 *±* 53.2 pmol mol ^−1^ at 19.8°C and 138.7 *±* 26.1 pmol mol ^−1^ at 25.0°C (error estimates denote 95% confidence interval). These results indicate that *Γ _COS_* increases with rising *T _leaf_*, suggesting a potential temperature dependence.

Although the empty-chamber regression effectively removed the baseline COS emission, some residual variability likely remained (RMSE = 6.9 pmol mol ^−1^ for [ *COS*] *_a_* and 2.73 pmol m ^−2^ s ^−1^ for *F _COS_*). To evaluate the potential influence of this unresolved background variability, we propagated these errors to the regression-derived *Γ _COS_.* When the uncertainty is included, the 95% confidence interval of *Γ _COS_* widened from *±* 53.2 to *±* 93.4 pmol mol ^−1^ at 19.8°C and from *±* 26.1 to *±* 48.9 pmol mol ^−1^ at 25.0°C.

The large uncertainty at 19.8°C indicates that the corresponding *Γ _COS_* value is statistically indistinguishable from zero. By contrast, the *Γ _COS_* measured at 25°C remains significant even when this is propagated and instrumental uncertainty is considered. Even accounting for the reported QCLS uncertainty of approximately 7.5 pmol mol ^−1^ for COS ( Kooijmans *et al.*, 2016), the *Γ _COS_* value at 25°C remains statistically robust.

**Figure 5.**
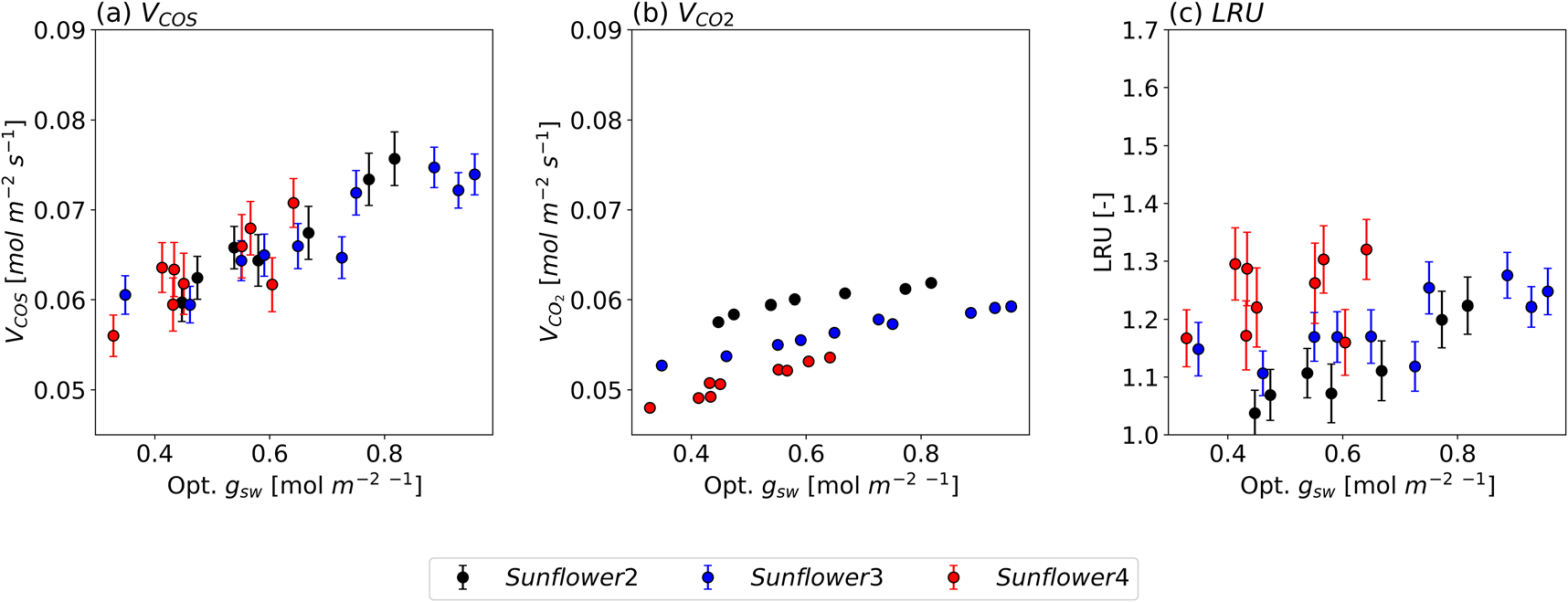
COS (a) and CO _2_ (b) deposition velocities and LRU changes (c) along the optimized *g _sw_* under leaf temperatures of approximately 25°C for three different sunflower plants, each represented in a different color. The circles and error bars represent the median values and standard deviations of the data points for their respective 150-second intervals.

***3.1.2 Responses to Stomatal Conductance (Experiment 2).*** Figure 5 illustrates the observed relationships of *V _COS_*, *V _CO _2__*, and LRU with optimized *g _sw_* for three sunflower plants. The observed values show that both *V _COS_* and *V _CO _2__* decrease with declining *g _sw_*, albeit at a slower rate at higher *g _sw_* values. Specifically, as *g _sw_* decreases from 1.0 mol m ^−2^ s ^−1^ to 0.4 mol m ^−2^ s ^−1^, *V _COS_* declines by approximately 40%, compared to a decline of about 10% in *V _CO _2__.* This difference persists even after accounting for the *g _sw_* ratio between COS and CO _2_ (1.21).

The greater sensitivity of *V _COS_* to changes in *g _sw_* is also reflected in the LRU, which consistently increases with *g _sw_* across all sunflowers. These findings support the hypothesis that COS uptake is more sensitive to variations in *g _sw_* compared to CO _2_ uptake. Meanwhile, slight differences in *V _COS_* and *V _CO _2__* values among individual sunflowers result in variations in LRU as well.

**Figure 6.**
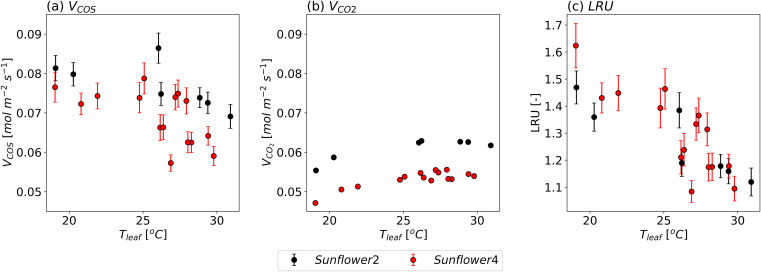
Same as Figure 5, but as a function of *T _leaf_* with maintained stomatal conductance at 0.6 *±* 0.1 mol m ^−2^ s ^−1^ (mean *±* standard deviation).

***3.1.3 Responses to Leaf Temperature (Experiment 3).*** Figure 6 shows the observed *V _COS_* and *V _CO _2__*, and LRU against *T _leaf_* for two sunflowers, with *g _sw_* held approximately constant. For all sunflowers, *V _COS_* decreases when temperatures exceed 25°C, whereas *V*_CO2_ increases with *T _leaf_* up to approximately 30°C. The absolute rate of decrease in *V _COS_* is larger than the rate of increase in *V _CO _2__.* Consequently, LRU decreases as *T _leaf_* increases. This trend can be attributed to the temperature responses of Γ _COS_ or/and the lower optimum temperature for CA than RuBisCO.

The temperature-dependent variations in *V _COS_* and *V _CO2_* also differ slightly between individual sunflower plants. While *V _COS_* exhibited relatively large observational errors, *V _CO2_* showed minimal errors. This suggests that while experimental uncertainties may influence *V _COS_*, the variations in both *V _COS_* and *V _CO _2__* are likely driven by micro-environmental differences, plant-to-plant variability, or other natural conditions, even within the same species grown in similar environments.

### 3.2 Model-assisted analysis

***3.2.1 Optimization Performance.*** We present the optimized results of model S2, which provides the best fit to the observations (Section 3.2.2).

Table 5 quantifies how well the optimized model estimates [COS] _out_, [CO _2_] _out_, and [H _2_O] _out_ compared to observations. The optimized model simulated the mole fractions of all three gases more accurately, reflected by mostly reduced values of *χ* ^2^, MSE, and MBE. Notably, the model biases were mitigated, as shown by the low posterior MBE values for [COS] _out_ (0.15 pmol mol ^−1^), [CO _2_] _out_ (– 0.01 μmol mol ^−1^), and [H _2_O] _out_ (0.07 mmol mol ^−1^). The *χ ^2^_poste_* values for [COS] _out_, [CO _2_] _out_, [H _2_O] _out_, and the state variables are 6.65, 1.68, 2.24, and 1.00, respectively. The large *χ ^2^_poste_* and RMSE for [COS] _out_ likely result from its relatively larger observational error.

**Table 5.** The statistical indices of the prior and posterior estimated [COS] _out_, [CO _2_] _out_, and [H _2_O] _out_ from model S2 compared to the observations with *χ* ^2^ changes of state term ( *x*). The units of RMSE and MBE of [COS] _out_ are pmol mol ^−1^, [CO _2_] _out_ is in μmol mol ^−1^, and [H _2_O] _out_ is mmol mol ^*−*1^.

Index	[COS] _**out**_		[CO _**2**_] _**out**_		[H _**2**_O] _**out**_		Background (State *x*)	
	Prior	Posterior	Prior	Posterior	Prior	Posterior	Prior	Posterior
χ ^2^	30.00	6.65	100.00	1.68	3.00	2.24	0.00	1.00
RMSE	16.71	7.93	3.63	0.71	0.30	0.45	-	-
MBE	11.77	0.15	-1.52	-0.01	-0.14	0.07	-	-

**Figure 7.**
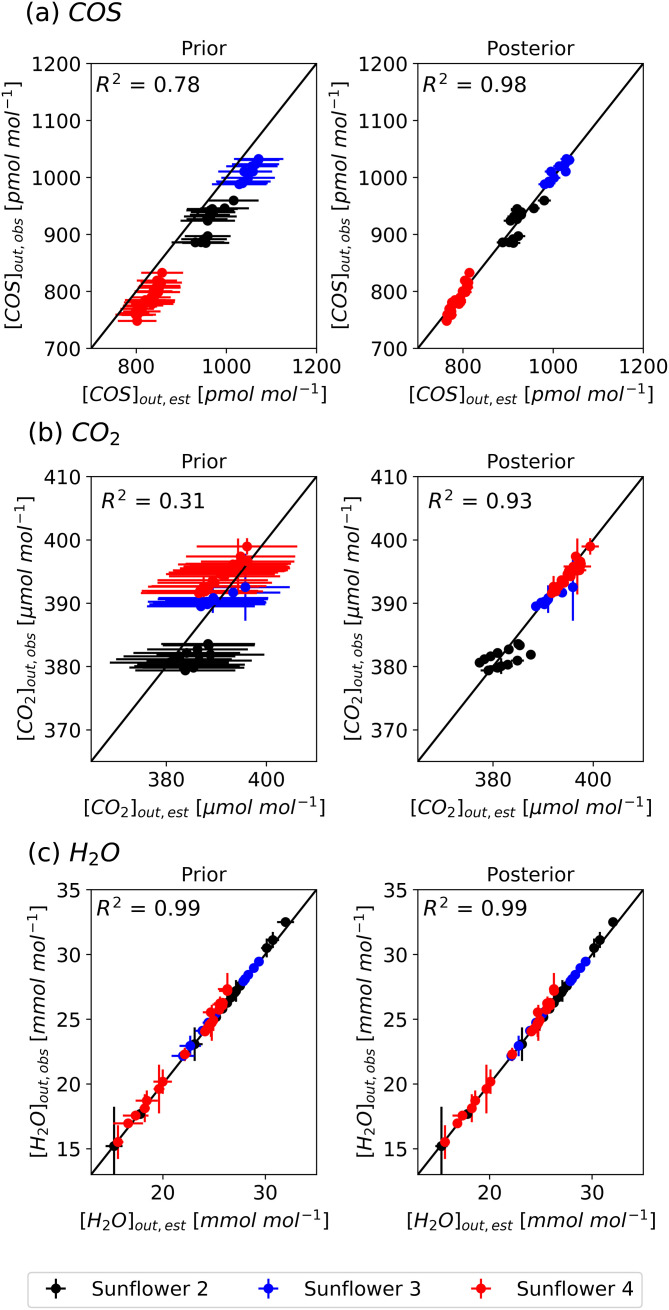
Scatter plots comparing the simulated (model S2) and observed atmospheric mole fractions of COS (a), CO _2_ (b), and H _2_O (c) with the prior parameters (left) and optimized parameters (right) with a 1:1 line indicating perfect agreement. The vertical error bars represent weighted observational errors, taken as the standard deviations of measurements, and the horizontal error bars represent prior errors (left) and posterior errors (right). Colors indicate different sunflower plants (black: Sunflower 2, blue: Sunflower 3, red: Sunflower 4).

To visualize these improvements, Figure 7 presents scatter plots comparing the simulated atmospheric mole fractions from the prior and posterior models to the observed values. The optimized atmospheric mole fractions exhibit improved agreement with the observed values across all sunflowers, improving the performance of the prior simulations. The optimized model explains the variability in the observations with high *R* ^2^ values from 0.93 to 0.99. The simulation errors, represented by the standard deviation of 500 forward simulations, were substantially reduced after optimization. Specifically for [COS] _out_, errors decreased from 47.4 pmol mol ^−1^ to 12.5 pmol mol ^−1^, for [CO _2_] _out_ from 10.8 μmol mol ^−1^ to 0.8 μmol mol ^−1^, and for [H _2_O] _out_ from 0.6 mmol mol ^−1^ to 0.3 mmol mol ^−1^. However, discrepancies remain, such as in Sunflower 2. Several posterior [COS] _out_ and [CO _2_] _out_ values of Sunflower 2 fall outside the weighted observational error range, likely due to measurement uncertainties.

In addition to reproducing the observations well, the optimization also reduced the parameter uncertainties. The optimized state values and their posterior errors, along with error reductions, are presented in Table 2. As expected, errors associated with all target parameters were significantly reduced, with an average error reduction of 48%. An error reduction of more than 92% was achieved for CO _2_ parameters ∆ *H _a,Rub_* and *V _max,Rub_.* Prior uncertainties of *T _eq,CA_*, *m*_COS_, and *V _max,CA_* were reduced by more than 30%, and the error reduction for *g _sw_* was about 43%.

Parameter values also shifted meaningfully after optimization. The optimized slope of *Γ _COS_* ( *m*_COS_, 22.4 pmol mol ^−1^ K ^−1^) is slightly increased to the initial setting (16.2 pmol mol ^−1^ K ^−1^). With the optimized value of *m*_COS_, the variable *T _eq,CA_* was adjusted to 39.7°C, and the values of *V _max,CA_* for each plant were optimized ranging from 0.189 to 0.230 mol m ^−2^ s ^−1^. These values increased significantly from their priors, 30.0°C and 0.125 mol m ^−2^ s ^−1^, indicating higher optimum temperatures and higher catalyzation velocities compared to the prior settings. The optimized *V _max,Rub_* ranged from 82.9 to 94.4 μmol m ^−2^ s ^−1^, while ∆ *H _a,Rub_* did not change substantially. The ratio of *V _max,CA_* to *V _max,Rub_* which is crucial for calculating CA activity from *V _max,Rub_* based on the assumption of a linear relationship, ranges from 2160 to 2770.

The optimized *g _sw_* also showed consistent behavior. Comparisons of the optimized mean *g _sw_* values of 0.64 mol m ^−2^ s ^−1^ with the prior values of 0.62 mol m ^−2^ s ^−1^ reveal slight differences. Errors in *g _sw_* were reduced from 0.09 to 0.05 mol m ^−2^ s ^−1^. The optimized model yielded an average relative humidity within the leaf (RH _i_) of 98.82 *±* 0.04%. These results confirm that allowing for slight non-saturation in the intercellular airspace (through *g _m_,H _2_O*) had a negligible impact on the modeled fluxes, validating the assumption that the air within the intercellular airspace was effectively saturated under our experimental conditions. Simulations further confirmed that varying *g _mw_* (<10 mol m ^−2^ s ^−1^) did not alter the modeled fluxes or mole fractions, indicating that its effect was negligible under our experimental conditions.

**Figure 8.**
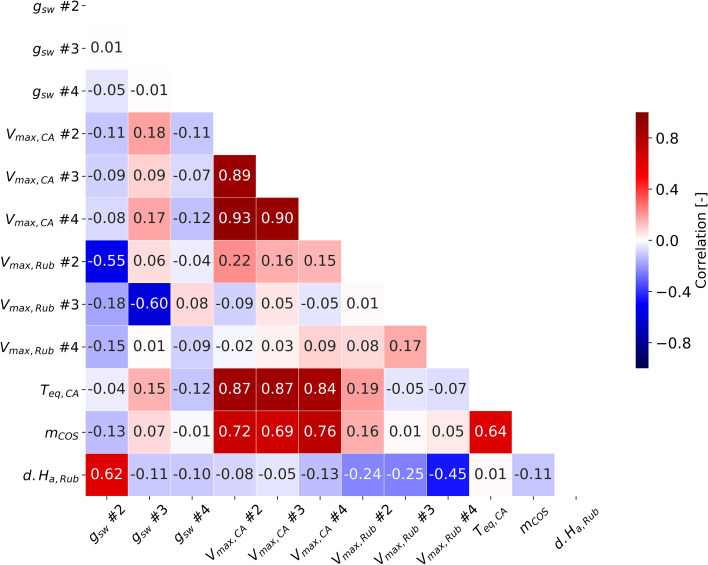
The covariance matrix for state variables. Regarding g _sw_, only the first data point of each sunflower is marked.

Figure 8 illustrates the posterior covariance matrix derived from Monte Carlo calculations. Although several state variables were highly correlated after optimization (values above 0.7), they were retained as state parameters because their simulation errors were substantially reduced. For instance, three variables of COS parameters ( *T _eq,CA_*, *m*_COS_, and *V _max,CA_*) were highly correlated, with values ranging from 0.69 to 0.87, as these parameters are all related to the mesophyll uptake of COS.

***3.2.2 COS Compensation Point (Hypothesis H1).*** Table 6 summarizes the changes in total cost ( *J _tot_*) between prior and posterior estimations for each *Γ _COS_* temperature function. Among the tested models, the lowest total cost was achieved with the linear model (S2), followed by the Arrhenius models S4 and S3, and the model without a compensation point (S1). These results confirm that incorporating *Γ _COS_* with a linear temperature function into the leaf exchange model provides the best agreement with observations, as it minimizes the overall cost function and improves model performance. However, the total cost difference between the S2 model and the S4 model was only 1 unit.

**Table 6.** Prior (Pri) and posterior (Poste) contributions to the cost function of the state term, H _2_O, CO _2_, and COS parts, depending on the prescription of *Γ _COS_* and its temperature dependence. *J _tot_* is the sum of costs for the states ( *J _bg_*), H _2_O ( *J*_H _2_O_), CO _2_ ( *J*_CO _2__), and COS ( *J*_COS_).

No.	Description of *Γ*_*COS*_		Cost							
			*J*_*bg*_	*J*_H _2_O_		*J*_CO _2__		*J*_COS_		*J*_*tot*_
	Function	Based T [ * °C*]	Post	Pri	Post	Pri	Post	Pri	Post	Post
S1	*Γ _COS_* = 0	-	54	144	97	4800	80	2437	349	580
S2	Linear	16.4	57		107		81	1440	320	564
S3	Arrhenius	19.8	59		100		80	2634	335	574
S4		25.0	61		106		80	2634	319	565

**Figure 9.**
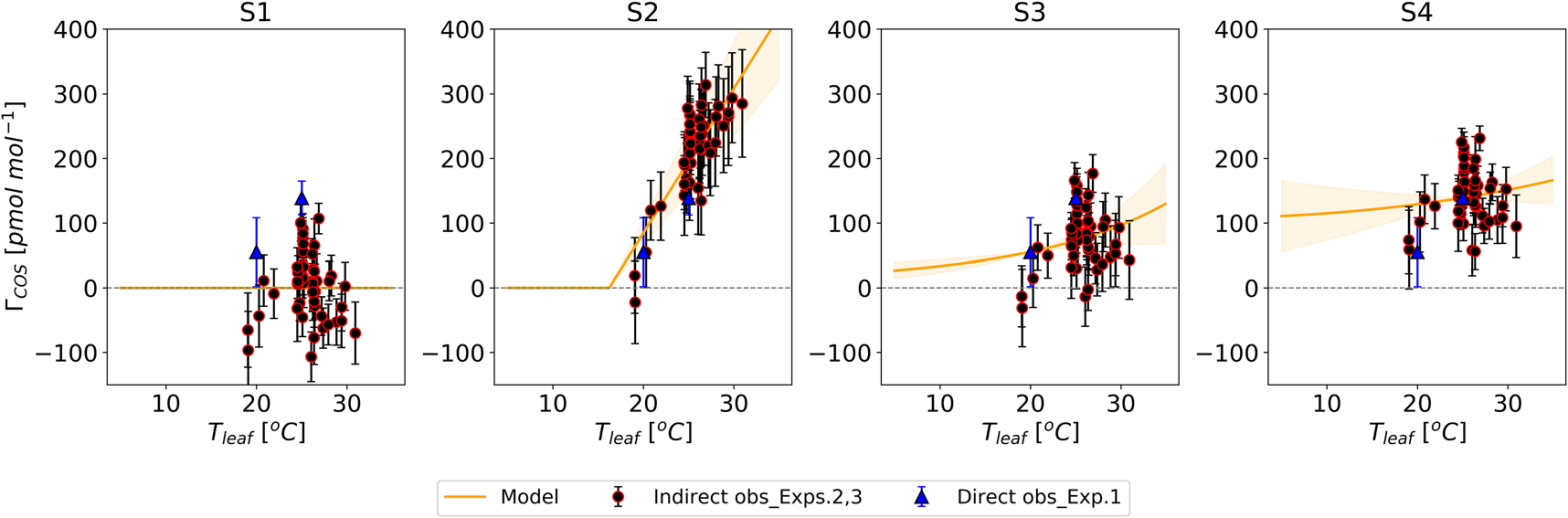
Posterior temperature functions for Γ _COS_ from S1 to S4 models, shown with orange lines and error ranges from 200 Monte Carlo simulations. These are compared with Γ _COS_ values from Experiment 1 (blue triangles, regression-derived from two temperature points) and Experiments 2 and 3 (black circles with red edges, derived indirectly). Regression-derived Γ _COS_ measurements are 55.0 ± 53.2 pmol mol ^−1^ at 19.8°C and 138.7 ± 26.1 pmol mol ^−1^ at 25.0°C, detailed in Section 3.1.1). Indirectly derived values are averages with standard deviation error bars, calculated from parameters optimized through 200 Monte Carlo simulations (See Section 2.5.).

To further evaluate the performance of these models, Figure 9 compares estimated *Γ _COS_* with two types of observation-based values across temperatures. The first type is regression-based measurements from 15 data points at two temperatures in Experiment 1 (blue triangles; see Figure 4). The second type is indirectly derived *Γ _COS_* in Experiments 2 and 3.

The optimized S2 model best captures the *Γ _COS_* measurements derived from both regression and indirect methods, compared to other models. Specifically, the S2 model achieved the lowest RMSE with the indirect derived *Γ _COS_* (39 pmol mol ^−1^). The RMSE values for the other models were: S1 = 50 pmol mol ^−1^, S3 = 49 pmol mol ^−1^, and S4 = 41 pmol mol ^−1^, respectively.

**Figure 10.**
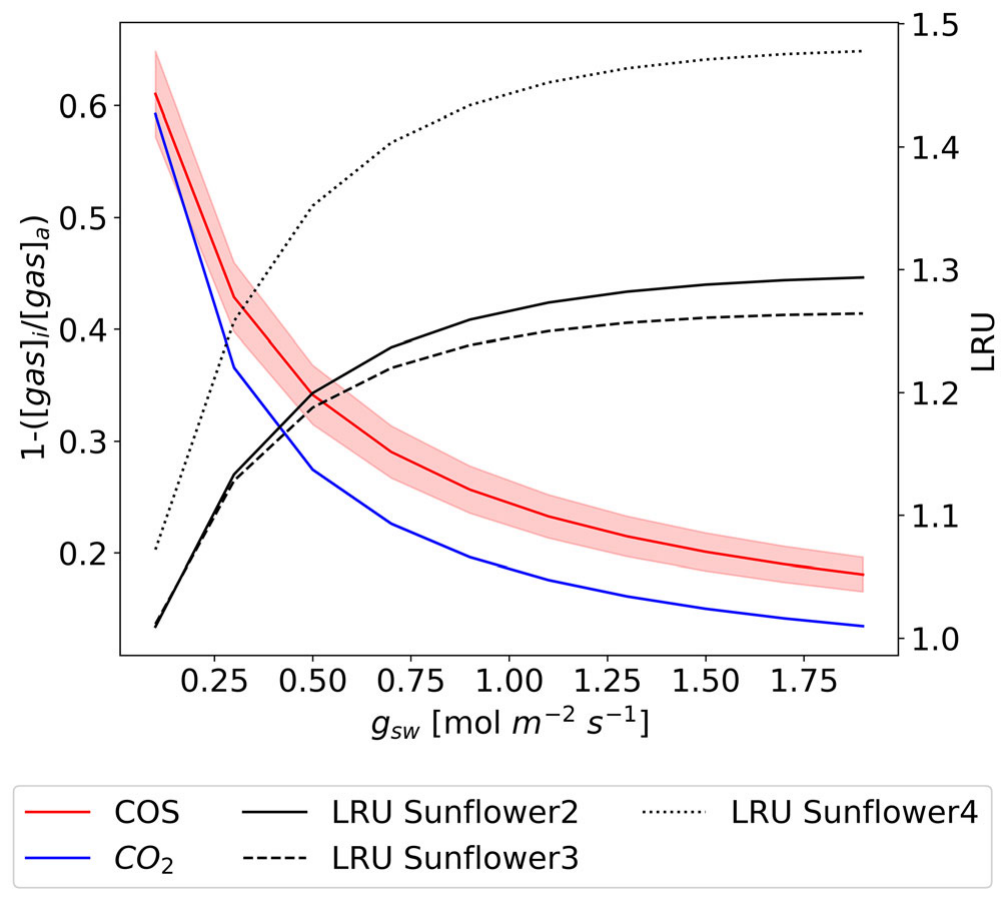
Simulated changes in (1 – [ *gas*] *_i_*/[ *gas*] *_a_*) for COS (red) and CO _2_ (blue) averaged among sunflowers 2, 3, and 4, with LRU (black, secondary y-axis) as a function of *g _sw_* each sunflower (Results from the S2 model). The lines represent averages, and the filled areas indicate the standard deviation of 500 simulations using posterior error propagation.

***3.2.3 Response to stomatal conductance (Hypothesis H2).*** In Experiment 2, we confirmed that *V _COS_* responds more strongly than *V _CO2_* at low *g _sw_* values. To further interpret this behavior mechanistically, we explore how changes in deposition velocity due to *g _sw_* are influenced by interactions with mesophyll conductance. We calculated AFR values (1 –[ *gas*] *_i_*/[ *gas*] *_a_*) for both gases using the optimized S2 model, as these values cannot be directly measured ( Figure 10). Simulations were performed at a constant *T _leaf_* of 25°C.

The model results indicate that for both gases, AFR decreases as *g _sw_* increases, which provides support for Hypothesis 2. However, contrary to Hypothesis 2, which assumes that [COS] _i_ is nearly zero thus resulting in AFR for COS close to 1, the actual modeled values range from 0.2 to 0.6 depending on *g _sw_.* This deviation indicates that [COS] _i_ is not negligible and suggests a dynamic COS consumption within the leaf that does not bring [COS] _i_ close to zero.

The model also reveals a distinct LRU pattern, showing sharp increases at low *g _sw_* values followed by stabilization at higher *g _sw_*. This response is consistent with observations from Experiment 2, where LRU exhibited similar trends in response to *g _sw_.* These results underscore the differing sensitivities of COS and CO _2_ uptake to *g _sw_*, as reflected in both experimental and modeled data.

**Figure 11.**
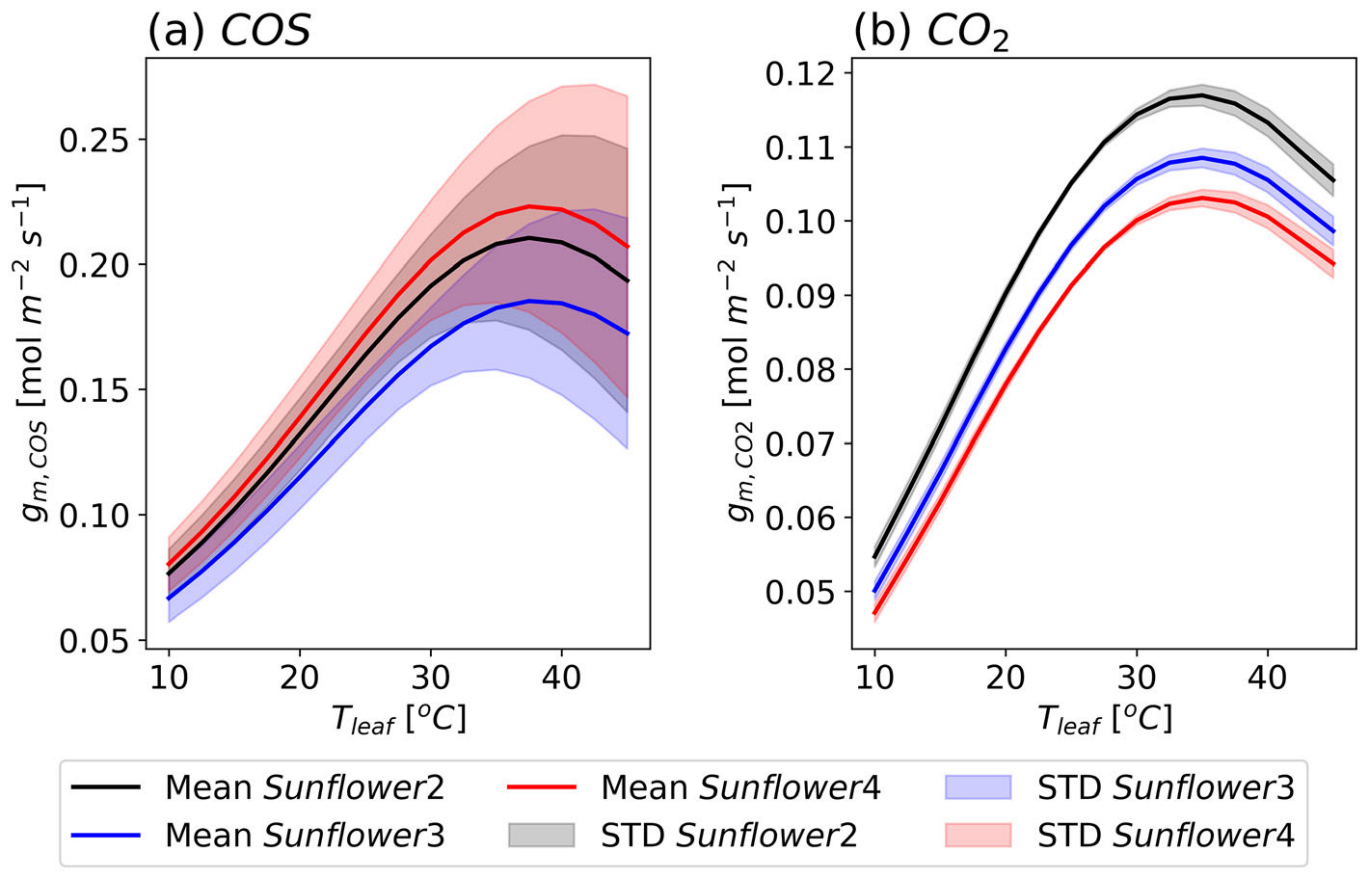
Modeled response of mesophyll conductances ( *g _m_*) to leaf temperatures ( *T _leaf_*) for COS (a) and CO _2_ (b) with three sunflowers (black: sunflower 2, blue: sunflower 3, red: sunflower 4, results from the S2 model). The lines represent averaged values, and the filled areas indicate the standard deviation from 500 simulations using posterior error propagation.

***3.2.4 Response to leaf temperature (Hypothesis H3).*** To investigate the temperature function of enzyme activity separated from *Γ _COS_* in *V _COS_*, we calculated *g _m,COS_* and *g*_*m,CO*_2__ using the optimized model S2. Figure 11 shows the temperature dependency of *g _m,COS_* and *g*_*m,CO*_2__ for three sunflowers. The magnitudes of these dependencies vary among plants due to differences in optimized *V _max_* values. The optimum temperature for *g _m,COS_* is around 35 to 40°C, while for *g*_*m,CO*_2__ it is approximately 35°C, which contradicts Hypothesis 3, proposing a lower optimum temperature for *g _m,COS_* than for *g*_*m,CO*_2__.

The observed decline in *V _COS_* above 25°C in Experiment 3 ( Figure 6a) is explained by the model-predicted impact of *Γ _COS_* on COS uptake. While the optimum temperature for *g _m,COS_* is approximately 35 to 40°C, the influence of temperature-dependent *Γ _COS_* results in a much lower optimum temperature for *V _COS_*, around 20°C. This is significantly lower than the optimum temperature for *V _CO _2__*, which is approximately 30°C.

Overall, the model results suggest that the temperature dependence of *Γ _COS_* plays a critical role in shaping the distinct responses of *V _COS_* and *V*_*COS*_2__.

## 4. Discussion

This study aimed to investigate how leaf COS and CO _2_ fluxes respond differently to environmental changes and to identify which internal processes within leaves govern these differences. Laboratory experiments were conducted under controlled conditions to isolate the effects of stomatal conductance and temperature. However, to provide more insights in the internal processes that control COS exchange, we developed and optimized a coupled CO _2_–COS–H _2_O conductance model to mechanistically interpret the measurement data. Although the model involves some simplified representations of internal processes, these simplifications are minor and do not affect the overall interpretation. In this section, we discuss the uncertainties in both observations and model representations, the physiological implications of the observed COS compensation points, and their broader significance for biochemical parameters and the global COS budget.

### 4.1 Uncertainties in measurements and models

Before discussing measurement uncertainties, we emphasize that potential leakage effects were minimized in our experimental setup. Through improvements in our experimental setup, especially with the LI-6800 system incorporating new gasket materials (Advanced Polymer, Polyethylene, and Neoprene) and optimized flow paths, we minimized the gas leakage issues prevalent in previous systems like the LI-6400. No significant pressure differentials were observed during experiments, supporting the effectiveness of these enhancements. Additionally, maintaining the cuvette under slight positive pressure reduces the risk of inward leakage.

Nevertheless, several experimental limitations remain. First, the temperature range explored in this study was relatively restricted. Our optimization was based on data from Experiments 2 and 3, conducted at temperatures ranging from 19.0°C to 29.8°C. Therefore, additional experiments across a broader temperature range are necessary to confirm the existence and assess the temperature dependence of *Γ _COS_.*

The choice of elevated COS concentration in the cuvette also introduces interpretative considerations. In our experiment, we increased the COS mole fraction in the cuvette to approximately 1000 pmol mol ^−1^, significantly above natural ambient levels, to enhance signal detection and reduce noise. The primary purpose of our empty chamber experiments was to eliminate the effects of emissions from chamber materials. Conducting these tests at similarly high mole fractions also helped mitigate any emissions that could arise from this higher mole fraction setting.

This high COS mole fraction possibly contributed to slightly higher AFR ( Figure 10) because the high ambient mole fraction may influence the internal concentration. [COS] _i_/[COS] _a_ values of about 0.63 ± 0.13 were observed under elevated ambient [COS] _a_ conditions ( Stimler *et al.*, 2010), aligning with our results. Stimler *et al.* (2010) also reported a linear relationship between [COS] _i_/[COS] _a_ and [COS] _a_, likely due to retro-diffusion from the leaves. Therefore, when applying our results to natural conditions ([COS] _a_ about 500 pmol mol ^−1^, the AFR may decrease. However, the high AFR is also possibly due to the influence of *Γ _COS_.* The simulated [CO _2_] _i_/[CO _2_] _a_ ranges from 0.4 to 0.9, consistent with values previously reported for C _3_ plants (0.5 – 0.8) ( Black & Jones, 1985; Seibt *et al.*, 2010; Tanner & Sinclair, 2015). Nevertheless, in Experiments 2 and 3, despite the high COS mole fractions, their consistent level throughout the experiments likely negated any impact on analyzing how *g _sw_* and temperatures relate to *V _COS_* and *V _CO _2__.*

In addition to the experimental limitations discussed above, a practical limitation arises from the mole fraction range used for model optimization. Model parameters were optimized using data obtained under relatively high COS mole fraction conditions (~1000 pmol mol ^−1^) to ensure stable and precise flux measurements, whereas *Γ _COS_* was determined from experiments conducted at lower inflowing concentrations (100–600 pmol mol ^−1^). This discrepancy in concentration ranges may introduce some uncertainty when extrapolating parameters between optimization and compensation-point estimation. Nevertheless, the model formulation remains internally consistent, and the derived parameters are valid for interpreting the relative temperature-dependent behavior of COS exchange. Future experiments targeting near-ambient COS mole fraction levels will be valuable to further validate the applicability of these optimized parameters and to evaluate potential retro-diffusion effects under more natural conditions.

From a modeling perspective, only the enzyme CA is considered in determining the mesophyll conductance of COS. However, other enzymes, such as RuBisCO and phosphoenolpyruvate carboxylase (PEP-C), are known to also catalyze COS uptake ( Lorimer & Pierce, 1989; Protoschill-Krebs & Kesselmeier, 1992). The contribution of RuBisCO to COS uptake is relatively minor compared to CA ( Protoschill-Krebs *et al.*, 1996), and its activity has not yet been quantified.

An additional source of uncertainty arises from the simplified representation of COS mesophyll conductance in our model. While mesophyll diffusion was not explicitly parameterized, it was implicitly represented through the temperature-dependent CA activity function ( Cho *et al.*, 2023) that integrates both diffusional and biochemical processes. Nevertheless, this implicit treatment may not fully capture variations in diffusional resistance under different environmental or structural conditions, and thus its influence on the temperature dependence of COS uptake cannot be completely excluded. Future modeling studies incorporating explicit mesophyll diffusion parameterization—beyond the current CA-based formulation—would help to disentangle the relative contributions of diffusional and biochemical controls, as well as potential COS emissions from leaves, in regulating COS exchange.

Beyond these structural uncertainties in the model, it is important to note that the conductance model was designed as an exploratory diagnostic tool. Its purpose was to integrate the coupled behavior of CO _2_, COS, and H _2_O exchange and to mechanistically interpret the observed fluxes. Despite its simplifications in not considering minor features, such as outlet mass flow change and boundary ternary effect, this framework represents the first attempt to jointly optimize gas exchange for the three gases, thereby providing a process-based means to independently estimate the COS compensation point. Overall, this approach highlights the potential of combined gas-exchange measurement and modeling to advance our understanding of COS biogeochemistry.

Taken together, the remaining uncertainties in both COS measurements and model structure require careful interpretation. Concerning Hypothesis 1, Model S2 was selected as the best model due to its minimal posterior costs and lowest RMSE with observation-based *Γ _COS_*, despite higher H _2_O and background costs compared to S1, as shown in Table 6. Furthermore, the minor differences in posterior costs and RMSE with indirectly derived *Γ _COS_* between S2 and S4 suggest that S4 is a viable alternative. The strong correlation between the temperature dependence of *Γ _COS_* ( *m*_COS_) and *V _max,CA_* complicates the precise and independent determination of these two parameters. Although S2 and S4 employ different temperature-dependent formulations of *Γ _COS_*, both model structures consistently support the presence of a COS compensation point.

Accordingly, the exact value and temperature dependence of *Γ _COS_* remain uncertain, but including a compensation point in the models, as in models S2 and S4, significantly improves their alignment with observations. Even if S4 were to better represent the true underlying mechanism, both model structures indicate an increase in *Γ _COS_* with temperature, although the optimum temperature derived under Hypothesis 3 may shift slightly depending on the assumed functional form of the temperature response of CA activity.

### 4.2 Possible causes of a COS compensation point

COS emission can occur due to leaf stress induced by external factors during experiments. To minimize the risk of mechanical damage or stress, the chamber was gently attached, and leaves were inspected post-experiment for physical damage such as bruising, desiccation, or discoloration. No visible damage was observed, and the limited measurement duration further reduced the likelihood of stress-induced COS emissions.

Previous studies on COS leaf exchange experiments have reported positive *Γ _COS_* values, but often concluded these are statistically indistinguishable from zero due to high variability. For example, Stimler *et al.* (2010) observed a *Γ _COS_* of 60.7 pmol mol ^−1^ at 25°C, and attributed it to potential retro-diffusion under high COS mole fractions. Similarly, our *Γ _COS_* at 20.0°C (55.0 ± 53.2 pmol mol ^−1^) was statistically insignificant, whereas a significant *Γ _COS_* (138.7 ± 26.1 pmol mol ^−1^) was detected at 25°C. Considering that our experiments, like those of Stimler *et al.* (2010), were conducted under relatively high COS mole fractions, this pattern suggests that higher temperature may enhance diffusion-driven feedbacks. It should also be noted that our COS model does not explicitly account for mesophyll diffusion conductance, and thus part of the apparent emission function could be interpreted as an effect of internal diffusion-related mechanisms (e.g., retro-diffusion) within the leaf.

In addition to retro-diffusion, temperature-induced stress could also contribute to the observed COS compensation point. Previous studies, typically conducted below 25°C, did not assess temperature dependence. Our modeling revealed a linear relationship between *Γ _COS_* and temperature, consistent with Gimeno *et al.* (2017), who reported higher *Γ _COS_* in mosses at elevated temperatures. Although no visible stress was observed in our plants, temperature stress may have influenced *Γ _COS_.* Gimeno *et al.* (2017) suggested that COS emissions could result from protein degradation under stress, releasing COS through the breakdown of sulfur-containing amino acids. This highlights the role of temperature-driven biochemical pathways in shaping *Γ _COS_* values and supports the inclusion of *Γ _COS_* in leaf gas exchange models.

Beyond temperature-driven stress, differences in species physiology also contribute to *Γ _COS_.* Mosses studied by Gimeno *et al.* (2017) exhibited higher *Γ _COS_* values than in vascular plants, likely due to enzymatic activity of associated fungi and bacteria. While microbial activity is not directly applicable to vascular plants, it underscores the importance of species-specific factors in determining COS emissions. In vascular plants, rapeseed is known to produce COS due to its high sulfur content, with *Γ _COS_* exceeding 100 pmol mol ^−1^ during ripening and senescence ( Belviso *et al.*, 2022). However, no experiments have previously been conducted to detect *Γ _COS_* in sunflowers.

Previous studies (e.g., Maseyk *et al.*, 2014; Belviso *et al.*, 2022) reported that COS emissions occur during the ripening and senescence phases, indicating that senescence-related processes could explain COS emissions rather than active uptake from living tissues. In Maseyk *et al.* (2014) part of these emissions may have originated from the soil, whereas Belviso *et al.* (2022) provided strong evidence for plant-derived senescence emissions. Additionally, Kesselmeier and Merk (1993) reported *Γ _COS_* values ranging from 57 to 328 pmol mol ^−1^ in rapeseed and corn during flowering. In our study, measurements were conducted during the flowering phase, which could explain the observed COS emissions, although the exact source remains unclear. Sulfur emissions during senescence have been linked to the translocation of sulfur-containing amino acids and sulfur regulation in plant cells ( Rennenberg, 1991).

Apart from biological factors, methodological advances may explain the discrepancies between our results and previous studies. We employed the Li-6800 system, which offers improved measurement accuracy, and evaluated an optimized conductance model incorporating temperature-dependent *Γ _COS_.* Our findings confirm that all observations support an increase in *Γ _COS_* with temperature.

Taken together, the existence of *Γ _COS_* and its increase at higher temperatures may result from a combination of the factors discussed above. However, our current formulation of *Γ _COS_* is expressed solely as a temperature-dependent function and does not explicitly include retro-diffusion effects. As a result, while the observed increase in *Γ _COS_* with temperature is consistent with thermally driven processes, the potential contribution of internal diffusion feedbacks—particularly under elevated COS mole fractions—cannot be fully excluded.

At present, reliable measurements can only be obtained under relatively high COS mole fractions, which limits our ability to fully constrain *Γ _COS_* dynamics near ambient conditions. Improving detection precision at low COS concentrations and incorporating explicit representations of internal diffusion in future models would enable a more direct evaluation of whether *Γ _COS_* variability originates from retro-diffusion or from physiological and biochemical processes. Moreover, extending such measurements to different phenological stages and species, and exploring interactions with microbial associations and environmental stressors, will be essential for developing a more mechanistic understanding of COS exchange in plants.

### 4.3 Impact of COS compensation point on *V _max,CA_*

When *Γ _COS_* was explicitly considered in the optimization, the optimized ratio between *V _max,CA_* and *V _max,Rub_* ranges from 2160 to 2770, which is higher than previously obtained values of approximately 1200 from laboratory measurements with C _3_ plants ( Berry *et al.*, 2013). Kooijmans *et al.* (2021) reported an average ratio of 1616 *±* 562 based on field observations in summer months. In contrast, when *Γ _COS_* was excluded (model S1), the optimized *V _max,CA_* became smaller, resulting in ratios of 1197–1327, closely matching the values reported in previous studies. Thus, models that incorporate *Γ _COS_* result in higher optimized values of *V _max,CA_* and consequently to higher ratios between *V _max,CA_* and *V _max,Rub_*, indicating a higher maximum catalyzation velocity of CA.

### 4.4 Consequences for the COS global budget

Our evidence of decreasing *V _COS_* between 20 and 25°C ( Figure 6a) supports lower biosphere uptake in high temperature regions. The current COS budget has a missing source of roughly 400 GgS year ^−1^, mainly due to the large biosphere sink calculated by the Simple Biosphere model (SiB4) in the tropics ( Berry *et al.*, 2013). In warm tropical forests, biosphere uptake would be significantly reduced.

The presence of *Γ _COS_* could have implications for the global COS budget and the utilization of COS in estimating GPP, especially under conditions such as drought, where high temperatures and low humidity persist. Despite the uncertainty concerning *Γ _COS_* in our experiments, including *Γ _COS_* in our model better reproduced CO _2_ and COS observations. This suggests that incorporating *Γ _COS_* into global biosphere models could enhance the accuracy of COS flux estimates. Differences in temperatures and humidity responses between CO _2_ and COS should also be considered. To further improve the large-scale modeling, it is recommended to measure *Γ _COS_* and enzyme activity across diverse plant types, specifically tropical plants.

While our study focused on controlled conditions with a single species, larger variations would indeed be expected under natural conditions and across different species. Consequently, future research should therefore explore these factors in more diverse environmental settings to validate and generalize these findings.

### Ethics and consent

Ethical approval and consent were not required.

## Data availability

All datasets generated from the experiments are available on GitHub and have been archived on Zenodo under a CC-BY 4.0 license.

- DOI: [ https://doi.org/10.5281/zenodo.15489794] ( Cho *et al.*, 2025)

- GitHub: [ https://github.com/dkfkcho/COS_sunflower]

## Software availability

- Source code available from: [ https://github.com/dkfkcho/COS_sunflower]

- Archived software available from: [ https://doi.org/10.5281/zenodo.15489794] ( Cho *et al.*, 2025)

- License: MIT License
