# Supplementary material for: Leaf chamber experiments on sunflowers indicate a temperature-dependent compensation point of carbonyl sulfide
Source: Open Res Eur. 2026 Apr 10;5:223. Originally published 2025 Aug 5. [Version 3] doi: 10.12688/openreseurope.20235.3 (PMC13168880; doi:10.12688/openreseurope.20235.3)
Supplement: Peer review history

# Reviewer report: Reviewer response for version 3

**DOI:** 10.21956/openreseurope.25391.r72166

**Published:** 2026-05-18

**Authors:** Jerome Ogee

**Affiliations:** 1 ISPA, Institut National de Recherche pour l'Agriculture l'Alimentation et l'Environnement Centre Nouvelle-Aquitaine Bordeaux (Ringgold ID: 113907), Villenave-d'Ornon, Aquitaine-Limousin-Poitou-Charentes, France; 2 Bordeaux Sciences Agro, Villenave d’Ornon, France

The authors thoroughly revised their manuscript since the first version I reviewed. I thank them for taking the time to address all my comments and follow some of my suggestions. I have no more comment on this version 3 and would recommend it for indexing.

Is the study design appropriate and does the work have academic merit?

Yes

Is the work clearly and accurately presented and does it cite the current literature?

Partly

If applicable, is the statistical analysis and its interpretation appropriate?

Partly

Are all the source data underlying the results available to ensure full reproducibility?

Yes

Are the conclusions drawn adequately supported by the results?

Partly

Are sufficient details of methods and analysis provided to allow replication by others?

Partly

Reviewer Expertise:

NA

I confirm that I have read this submission and believe that I have an appropriate level of expertise to confirm that it is of an acceptable scientific standard.

# Reviewer report: Reviewer response for version 3

**DOI:** 10.21956/openreseurope.25391.r72168

**Published:** 2026-05-12

**Authors:** Anam M. Khan

**Affiliations:** 1 Northern Arizona University (Ringgold ID: 3356), Flagstaff, Arizona, USA

I have no further comments. I approve of this manuscript.

Is the study design appropriate and does the work have academic merit?

Yes

Is the work clearly and accurately presented and does it cite the current literature?

Partly

If applicable, is the statistical analysis and its interpretation appropriate?

Yes

Are all the source data underlying the results available to ensure full reproducibility?

Yes

Are the conclusions drawn adequately supported by the results?

Yes

Are sufficient details of methods and analysis provided to allow replication by others?

Partly

Reviewer Expertise:

deposition velocity, stomatal conductance, surface-atmosphere exchange

I confirm that I have read this submission and believe that I have an appropriate level of expertise to confirm that it is of an acceptable scientific standard.

# Reviewer report: Reviewer response for version 2

**DOI:** 10.21956/openreseurope.23939.r66532

**Published:** 2026-02-04

**Authors:** Anam M. Khan

**Affiliations:** 1 Northern Arizona University (Ringgold ID: 3356), Flagstaff, Arizona, USA

The authors present interesting gas exchange data for COS and CO _2_ from sunflower. They demonstrate the existence of a significant COS compensation point at a temperature of 25C, and they find that sunflower emits COS at low ambient [COS] at this temperature. The comparison between the response of COS and CO _2_ leaf uptake to stomatal conductance is interesting. However, some clarifications are needed to ensure reproducibility, and the calculation of deposition velocity needs more clarification as well.

 Specific comments:

1. In the case of depositing a gas from ambient air to intercellular spaces, the deposition velocity (Vd) is a flux divided by the concentration gradient between the ambient air and the intercellular space, Vd = flux/(ambient [gas] – intercellular [gas]). For some gasses, it is assumed that intercellular [gas] is 0. Then, the Vd is simply flux / ambient [gas]. The assumption of intercellular [gas] = 0 does not hold for CO _2_, and the modeling in this study seems to suggest that it does not hold for COS either. The authors should describe the rationale used to calculate Vd in equation 2. Why is the flux being normalized by the [gas] _out_? The [gas] _out_ has no significance without knowing the [gas] _in_. The decrease in the concentration of the gas in the ambient air of the leaf chamber ([gas] _in_ – [gas] _out_) approximates the uptake by the plant material in the chamber. If the goal was to normalize the flux by ambient [gas], then [gas] _in_ approximates the ambient [gas] that the plant material was exposed to. The [gas] _out_ is just the [gas] after the uptake or emission has taken place in the chamber. There might be adequate reason for calculating Vd as it was calculated in equation 2, but it is not clear.
2. The growth conditions of the plants and the timings of measurements need to be explained if the experiment is going to be reproduced. What were the plants’ growing conditions? Which time of the day were gas exchange measurements taken?
3. The modeling reveals issues of model equifinality where two different model structures, S2 and S4, have a very similar agreement with observations. The authors briefly mention that S4 is a viable alternative to S2. Can they expand on which structure reflects the true mechanisms if both have very similar agreement with observations?

Minor comments:

1. Consider submitting manuscripts for peer review with line numbers.
2. The paragraph structure of the manuscript needs extensive editing.
3. Caption for Table 1 might need revision. It says, “designed to investigate responses of V _COS_ and V _CO2_ to g _s_ and g _m_,  respectively.” Was experiment 3 investigating the response to T _leaf_ or g _m_? I’m assuming T _leaf_ since that is what the table header and manuscript text says.

Is the study design appropriate and does the work have academic merit?

Yes

Is the work clearly and accurately presented and does it cite the current literature?

Partly

If applicable, is the statistical analysis and its interpretation appropriate?

Yes

Are all the source data underlying the results available to ensure full reproducibility?

Yes

Are the conclusions drawn adequately supported by the results?

Yes

Are sufficient details of methods and analysis provided to allow replication by others?

Partly

Reviewer Expertise:

deposition velocity, stomatal conductance, surface-atmosphere exchange

I confirm that I have read this submission and believe that I have an appropriate level of expertise to confirm that it is of an acceptable scientific standard, however I have significant reservations, as outlined above.

# Response

**Published:** 2026-03-17

**Authors:** Ara Cho

**Affiliations:** Meteorology and Air Quality, Wageningen University & Research, Wageningen, Gelderland, The Netherlands

The authors present interesting gas exchange data for COS and CO _2_ from sunflower. They demonstrate the existence of a significant COS compensation point at a temperature of 25C, and they find that sunflower emits COS at low ambient [COS] at this temperature. The comparison between the response of COS and CO _2_ leaf uptake to stomatal conductance is interesting. However, some clarifications are needed to ensure reproducibility, and the calculation of deposition velocity needs more clarification as well.   Specific comments:

1. In the case of depositing a gas from ambient air to intercellular spaces, the deposition velocity (Vd) is a flux divided by the concentration gradient between the ambient air and the intercellular space, Vd = flux/(ambient [gas] – intercellular [gas]). For some gasses, it is assumed that intercellular [gas] is 0. Then, the Vd is simply flux / ambient [gas]. The assumption of intercellular [gas] = 0 does not hold for CO _2_, and the modeling in this study seems to suggest that it does not hold for COS either. The authors should describe the rationale used to calculate Vd in equation 2. Why is the flux being normalized by the [gas] _out_? The [gas] _out_ has no significance without knowing the [gas] _in_. The decrease in the concentration of the gas in the ambient air of the leaf chamber ([gas] _in_ – [gas] _out_) approximates the uptake by the plant material in the chamber. If the goal was to normalize the flux by ambient [gas], then [gas] _in_ approximates the ambient [gas] that the plant material was exposed to. The [gas] _out_ is just the [gas] after the uptake or emission has taken place in the chamber. There might be adequate reason for calculating Vd as it was calculated in equation 2, but it is not clear.

  We thank the reviewer for this thoughtful question. Indeed, the difference between C _in _and C _out_ provides a measure of plant uptake within the chamber. As stated in the Methods section, deposition velocities were calculated using the outgoing mole fraction (C _out_). The rationale for this choice is the assumption of a well-mixed chamber, under which C _out_ is considered representative of the air mole fraction surrounding the experienced by the leaf. We therefore regard C _out_ as the most appropriate bulk mole fraction for normalizing the flux. We have clarified this explicitly in the manuscript immediately following Equation 2.   2.1.2. Experiments The measured V _COS_ and V _CO2_ were used to calculate the LRU (-), which was used to characterize differing response of COS and CO _2_ from Experiments 2 and 3 (Sandoval-Soto et al., 2005; Campbell et al., 2008) : ...Equation 2... where A _COS_ (pmol m ^−2^ s ^−1^) and A _CO2_ (μmol m ^−2^ s ^−1^) are assimilation rates of COS and CO _2_, respectively. These rates were normalized by outflowing concentrations [COS] _out_ and [CO _2_] _out_, which are assumed to be the concentrations the plants are exposed to due to the well-mixed conditions (see Sect. 2.2.1) 

 2. The growth conditions of the plants and the timings of measurements need to be explained if the experiment is going to be reproduced. What were the plants’ growing conditions? Which time of the day were gas exchange measurements taken?  

 We thank the reviewer for this comment. We have clarified both the growth conditions of the plant material and the timing of the gas exchange measurements in the Methods section to improve reproducibility. Sunflower plants were obtained from a local plant nursery (Evanthia, Maasdijk, the Netherlands) and cultivated in a greenhouse under a day/night temperature regime of 18–21 ℃ during the day and 15–18 ℃ at night, with ventilation applied above 20 ℃. Germination occurred at 21–24 ℃. In addition, we now specify that gas exchange measurements were scheduled to align with the plants’ photosynthetic rhythm and were conducted within the local daytime window between 10:00 and 17:00 (local time, the Netherlands), with each individual plant measured for a maximum duration of 5 daytime hours.  

 We measured deposition velocities of COS and CO₂ (V _COS_ and V _CO2_), i.e., fluxes normalized by mole fractions in air, using a leaf cuvette system. These experiments were conducted using sunflower plants (Helianthus annuus L. cv. ‘Sunsation’), which are C₃ photosynthesis type plants cultivated in a local plant nursery (Evanthia, Maasdijk, the Netherlands). Plants were cultivated in a greenhouse under a day/night temperature regime of 18–21 ℃ during the day and 15–18 ℃ at night, with ventilation applied above 20 ℃. Germination occurred at 21–24 ℃.  Each sunflower plant was used for a maximum of 5 daytime hours to minimize physiological stress caused by prolonged exposure to experimental conditions. Gas exchange measurements were scheduled to align with the plants’ photosynthetic diurnal rhythm and were conducted during the local daytime window between 10:00 and 17:00 (local time, the Netherlands). 

  

1. The modeling reveals issues of model equifinality where two different model structures, S2 and S4, have a very similar agreement with observations. The authors briefly mention that S4 is a viable alternative to S2. Can they expand on which structure reflects the true mechanisms if both have very similar agreement with observations?

  We agree that models S2 and S4 show similar performance and therefore cannot be uniquely distinguished based on the available data. Both model structures assume the presence of a COS compensation point, but differ in the steepness of its temperature dependence. While posterior costs were comparable, S2 consistently showed the lowest RMSE and most closely reproduced the observations from Experiment 1 (Fig. 2.9). Based on this overall performance across multiple metrics, S2 was therefore selected as the best-performing model in this study. We have expanded the Discussion to clarify this rationale and to note that, even if S4 were to better represent the true mechanism, the key conclusion of a temperature-dependent COS compensation point remains unchanged, although the exact optimum temperature associated with Hypothesis H3 may vary slightly.  

 4.1. Uncertainties in measurements and models Taken together, the remaining uncertainties in both COS measurements and model structure require careful interpretation. Concerning Hypothesis 1, Model S2 was selected as the best model due to its minimal posterior costs and lowest RMSE with observation-based Γ _COS_, despite higher H _2_O and background costs compared to S1, as shown in Table 2.6. Furthermore, the minor differences in posterior costs and RMSE with indirectly derived Γ _COS_ between S2 and S4 suggest that S4 is a viable alternative. The strong correlation between the temperature dependence of Γ _COS_ (m _COS_) and V _max,CA_ complicates the precise and independent determination of these two parameters. Although S2 and S4 employ different temperature-dependent formulations of Γ _COS_, both model structures consistently support the presence of a COS compensation point.   Accordingly, the exact value and temperature dependence of Γ _COS_ remain uncertain, but including a compensation point in the models, as in models S2 and S4, significantly improves their alignment with observations. Even if S4 were to better represent the true underlying mechanism, both model structures indicate an increase in Γ _COS_ with temperature, although the optimum temperature derived under Hypothesis 3 may shift slightly depending on the assumed functional form of the temperature response of CA activity. 

 Minor comments:

1. Consider submitting manuscripts for peer review with line numbers.

We thank the reviewer for this suggestion. The manuscript template from Open Research Europe does not support the inclusion of line numbers. We will contact Open Research Europe to clarify whether line numbers can be included within the provided manuscript template.  

  

1. The paragraph structure of the manuscript needs extensive editing.

We thank the reviewer for this comment. We carefully revisited the manuscript to improve paragraph structure and overall readability. In particular, Section 4.1 has been reorganized to more clearly separate experimental limitations from model-related uncertainties, and several long paragraphs were divided and streamlined to ensure that each paragraph addresses a single, well-defined topic. Transitions between experimental constraints and structural model assumptions were clarified to improve logical flow. Minor adjustments were also made in other sections to enhance coherence and reduce redundancy. We believe these revisions substantially improve the readability and structural clarity of the manuscript.  

  

1. Caption for Table 1 might need revision. It says, “designed to investigate responses of V _COS_ and V _CO2_ to g _s_ and g _m_,  respectively.” Was experiment 3 investigating the response to T _leaf_ or g _m_? I’m assuming T _leaf_ since that is what the table header and manuscript text says.

We thank the reviewer for pointing this out. The reviewer is correct that Experiment 3 investigated responses to leaf temperature (T _leaf_). While T _leaf_ was the manipulated variable, this experiment was designed to explore temperature-driven changes in mesophyll conductance (g _m_), for which T _leaf_ is a major controlling factor. We have revised the caption of Table 1 accordingly to clarify this distinction and ensure consistency with the table header and manuscript text.

 Table 1. Environmental conditions during the three experiments. The range of controlled and constant variables for each experiment is expressed as minimum–maximum and mean ± standard deviation, respectively. Experiment 1 (Sunflower 1) aimed to detect COS compensation points at two temperatures. Experiments 2 (Sunflower 2, 3, and 4) and 3 (Sunflower 2 and 4) were designed to investigate responses of V _COS_ and V _CO2 _to stomatal conductance (g _s_) and leaf temperature (T _leaf_), respectively, with T _leaf_ acting as a primary driver of mesophyll conductance (g _m_) . We controlled a designated variable in each experiment while other variables including light intensity, air flow rate, and mixing fan speed were kept constant.

# Reviewer report: Reviewer response for version 1

**DOI:** 10.21956/openreseurope.21895.r58703

**Published:** 2025-09-24

**Authors:** Max Berkelhammer

**Affiliations:** 1 University of Illinois Chicago, Chicago, USA

This paper presents a set of leaf chamber and modeling experiments to test fundamental and poorly constrained aspects of the leaf-level exchange of OCS.  The authors show evidence of a temperature-dependent compensation point and indicate how the differential effects of temperature and VPD on OCS and CO2 change the relative uptake of these two gases. Firstly, I’d like to commend the authors on difficult lab experiments that were well-designed and executed.  Secondly, the modeling experiments allowed the authors to clearly test three fundamental hypotheses. While the sources of uncertainty in the experiments are large, the manuscript raises a lot of interesting questions that open the door for interesting work to come. So while I support the publication of the manuscript, I do feel that the structure and style of the manuscript requires some careful considerations.

1. The paper is written in a strange style where many paragraphs seem to be one sentence and essentially like a bullet point. I am not sure if this was intentional or some artifact of the formatting. Nevertheless, paragraphs should be structured in a more standard format  where the reader knows the purpose of the paragraph from the opening sentence and the end of the paragraph sets up the next paragraph.  The issue is not just about following traditional writing structure but, as is, the flow of the paper is not great. I think the authors should consider improving the flow, removing some superfluous text and following standard formatting. That said, the individual sentences were generally well-written and there were barely any grammatical issues.
2. As I was reading the paper, I found myself thinking a lot about the Stimler experiments and how they compared. The authors bring in the details of the Stimler paper in the Discussion but this should be part of the Introduction. For example, the fact that Stimler also saw a compensation point at around pmol mol should be discussed in the beginning of the paper.  Furthermore, it would be interesting, if reasonable, to actually add some of the Stimler results to your figures so the authors can directly see how the results compare.
3. The authors note some ecosystem studies like those of Maseyk that suggest a compensation point or emission at high temperature but, I believe, these were emissions after the crops had senesced and were possibly from the soil.  Please clarify if they actually documented emissions from living wheat. 
4. Perhaps this was discussed in previous studies but is there any documentation here on how the uncertainty of the instrument varies with OCS concentration. At these low concentrations – around 100 pmol mol – how uncertain are the measurements?
5. Insufficient attention was paid to the potential importance of the temperature-dependent OCS uptake from the empty cuvette.  For example, at 25C, the uptake of OCS varies from 0 to -12.5.  This is a big range and obviously highly significant when considering the question of compensation points and very low fluxes at low [OCS] concentrations. When I see in Figure 4 that the uncertainty of the flux is about 5 pmol m-2 s-1 at 100 pmol mol-1, this seems discordant with such a potentially large range in fluxes from the empty chamber. I acknowledge that this may be dealt with in the statistics so I am not, per se, questioning the result but just asking for more careful deliberation about the potential impacts of the empty chamber flux.
6. The authors acknowledge this in the Discussion but I also found myself concerned by the fact that the experiments were optimized using observations conducted under conditions where [OCS] was an order of magnitude higher than the compensation point yet these simulations were used to study compensation point dynamics. I understand the operational reasons for this and I think the authors were transparent about this but it leaves quite a lot of room for future studies.

No page numbers.

 Line 1: “quantifies the _____ gross CO2….”  Should that “largest gross”?

 Top  of Page 5: “stomatal closure like the case”

 Page 10: The text following Equation 7 needs to be rewritten

Is the study design appropriate and does the work have academic merit?

Yes

Is the work clearly and accurately presented and does it cite the current literature?

Yes

If applicable, is the statistical analysis and its interpretation appropriate?

Yes

Are all the source data underlying the results available to ensure full reproducibility?

Yes

Are the conclusions drawn adequately supported by the results?

Partly

Are sufficient details of methods and analysis provided to allow replication by others?

Yes

Reviewer Expertise:

Atmosphere, Climate and Ecosystems

I confirm that I have read this submission and believe that I have an appropriate level of expertise to confirm that it is of an acceptable scientific standard, however I have significant reservations, as outlined above.

# Response

**Published:** 2025-12-03

**Authors:** Ara Cho

**Affiliations:** Meteorology and Air Quality, Wageningen University & Research, Wageningen, Gelderland, The Netherlands

1. The paper is written in a strange style where many paragraphs seem to be one sentence and essentially like a bullet point. I am not sure if this was intentional or some artifact of the formatting. Nevertheless, paragraphs should be structured in a more standard format where the reader knows the purpose of the paragraph from the opening sentence and the end of the paragraph sets up the next paragraph. The issue is not just about following traditional writing structure but, as is, the flow of the paper is not great. I think the authors should consider improving the flow, removing some superfluous text and following standard formatting. That said, the individual sentences were generally well-written and there were barely any grammatical issues.

 We sincerely thank the reviewer for this valuable comment and fully agree that the previous version of the manuscript suffered from an overly fragmented paragraph structure, which reduced the overall readability and logical flow. To address this issue, we thoroughly revised the manuscript to ensure that each paragraph now follows a clear logical structure: an opening statement that introduces the main idea, supporting sentences that develop the argument, and a concluding sentence that transitions smoothly to the next paragraph. In particular, we merged overly short bullet-like paragraphs and added linking sentences.As a result, we hope that the manuscript now reads more easily, and that the connection between experimental observations, model results, and their broader implications is clearer.

 Because these revisions were made throughout the manuscript to improve the overall readability and flow, we did not mark them individually in the main text.  

 2. As I was reading the paper, I found myself thinking a lot about the Stimler experiments and how they compared. The authors bring in the details of the Stimler paper in the Discussion but this should be part of the Introduction. For example, the fact that Stimler also saw a compensation point at around pmol mol should be discussed in the beginning of the paper.  Furthermore, it would be interesting, if reasonable, to actually add some of the Stimler results to your figures so the authors can directly see how the results compare.

 We thank the reviewer for this helpful suggestion. In the revised manuscript, we have incorporated the key details of the Stimler et al. (2010) study into the Introduction, providing a clearer background and establishing the context for our investigation of the COS compensation point. Specifically, we now mention that Stimler et al. observed a compensation point of 60.7 pmol mol⁻¹, despite it being statistically indistinguishable from zero, and interpreted it as a diffusional feedback under high COS mole fractions. This helps frame our study within prior findings and emphasizes the motivation for exploring temperature-dependent behavior in vascular plants. However, we have chosen not to include the Stimler et al. (2010) results in our figures, because direct graphical comparison is not scientifically consistent across species and experimental conditions. Stimler et al. (2010) investigated *Salvia indigo spires*, while Kesselmeier and Merk (1993) reported Γ _COS_ values of 90 pmol mol⁻¹ for *rapeseed* and 144 pmol mol⁻¹ for *corn*, and our study focused on *Helianthus annuus* (sunflower). These species differ substantially in leaf anatomy, sulfur metabolism, and stomatal physiology, leading to large inherent variations in COS compensation points. Moreover, experimental setups (e.g., light intensity, humidity, chamber design, etc) and analytical precision differed between studies, making a direct quantitative comparison potentially misleading.

 Γ _COS_ have been reported in a few vascular plants, algae, crops, and lichen fields ( Belviso et al., 2022; Geng & Mu, 2004; Goldan et al., 1988; Kesselmeier & Merk, 1993; Kuhn & Kesselmeier, 2000; Maseyk et al., 2014). These measured Γ _COS_ values are lower than typical atmospheric COS mole fractions ( ≈ 500 pmol mol −1), which is why net COS uptake is observed. Stimler et al. (2010) also observed a Γ _COS_ of 60.7 pmol mol −1, though statistically indistinguishable from zero, and interpreted it as a possible diffusional feedback under high COS mole fractions. More recently, Gimeno et al. (2017) demonstrated temperature-dependent COS emissions in nonvascular bryophytes, suggesting that biochemical processes such as protein degradation could contribute to COS release at elevated T _leaf_. However, Γ _COS_ of vascular plants and its temperature dependence remain poorly constrained. We therefore hypothesize that the observed COS uptake results from the coexistence of uptake and production processes, with Γ _COS_ exhibiting temperature dependence. 

****4.2. Possible causes of a COS compensation point** **>** Previous studies on COS leaf exchange experiments have reported positive *Γ _COS_* values, but often concluded these are statistically indistinguishable from zero due to high variability. For example, Stimler et al. (2010) observed a *Γ _COS_* of 60.7 pmol mol ^−1^ at 25 °C and attributed it to potential retro-diffusion under high COS mole fractions. Similarly, our *Γ _COS_* at 20.0 °C (55.0 ± 53.2 pmol mol *^−^* ^1^) was statistically insignificant, whereas a significant *Γ _COS_* (138.7 ± 26.1 pmol mol *^−^* ^1^) was detected at 25 °C. Considering that our experiments, like those of Stimler et al. (2010), were conducted under relatively high COS mole fractions, this pattern suggests that higher temperature may enhance diffusion-driven feedbacks. It should also be noted that our COS model does not explicitly account for mesophyll diffusion conductance, and thus part of the apparent emission function could be interpreted as an effect of internal diffusion-related mechanisms (e.g., retro-diffusion) within the leaf.   The authors note some ecosystem studies like those of Maseyk that suggest a compensation point or emission at high temperature but, I believe, these were emissions after the crops had senesced and were possibly from the soil.  Please clarify if they actually documented emissions from living wheat.  We thank the reviewer for this insightful comment. Both Maseyk et al. (2014) and Belviso et al. (2022) reported transitions from COS uptake to COS emission during the ripening and senescence phases of wheat (and rapeseed). These emissions are therefore likely associated with senescence-related processes rather than active uptake or release from photosynthetically functioning tissues.  Maseyk et al. (2014) additionally noted that part of the observed emissions could originate from the soil, whereas Belviso et al. (2022) provided stronger evidence for plant-derived emissions during senescence. Based on this, we clarify this point and discuss these studies only in the context of senescence-related ecosystem COS emissions.**

****4.2. Possible causes of a COS compensation point** **>** Previous studies (e.g., Maseyk et al., 2014; Belviso et al., 2022) reported that COS emissions occur during the ripening and senescence phases, indicating that senescence-related processes could explain COS emissions rather than active uptake from living tissues. In Maseyk et al. (2014) part of these emissions may have originated from the soil, whereas Belviso et al. (2022) provided strong evidence for plant-derived senescence emissions. Additionally, Kesselmeier and Merk (1993) reported Γ _COS_ values ranging from 57 to 328 pmol mol ^−1^ in rapeseed and corn during flowering.   Perhaps this was discussed in previous studies but is there any documentation here on how the uncertainty of the instrument varies with OCS concentration. At these low concentrations – around 100 pmol mol – how uncertain are the measurements? To our knowledge, specific evaluations of QCLS precision as a function of COS mole fraction at low level have not been explicitly reported in the literature. However, previous studies have demonstrated a typical precision of approximately 7.5 pmol mol⁻¹ for COS at low ambient mole fractions (Kooijmans et al., 2016). We have now added this information to the Results section (Section 3.1.1) and included a quantitative uncertainty analysis that propagates both instrumental and regression-derived errors (from empty chamber) into the estimation of Γ _COS_. This addition clarifies that, even when accounting for this uncertainty, the Γ _COS_ value observed at 25 °C remains statistically robust.**

****<** **3.1.1. COS Compensation Point (Experiment 1** **)>****

**We quantified the regression-based Γ _COS_ using a linear function at two temperatures: Γ _COS_ was 55.0 ± 53.2 pmol mol ^−1^ at 19.8 °C and 138.7 ± 26.1 pmol mol ^−1^ at 25.0 °C (error estimates denote 95 % confidence interval). These results indicate that Γ _COS_ increases with rising T _leaf_, suggesting a potential temperature dependence. Although the empty-chamber regression effectively removed the baseline COS emission, some residual variability likely remained (RMSE = 6.9 pmol mol ^−1^ for [COS] _a_ and 2.73 pmol m ^−2^ s ^−1^ for F _COS_). To evaluate the potential influence of this unresolved background variability, we propagated these residual errors to the regression-derived Γ _COS_. When the uncertainty is included, the 95% confidence interval of Γ _COS_ widened from ± 53.2 to ± 93.4 pmol mol ^−1^ at 19.8 °C and from ± 26.1 to ± 48.9 pmol mol ^−1^ at 25.0 °C. The large uncertainty at 19.8 °C indicates that the corresponding Γ _COS_ value is statistically indistinguishable from zero. By contrast, the Γ _COS_ measured at 25 °C remains significant even when this propagated and instrumental uncertainty is considered. Even accounting for the reported QCLS uncertainty of approximately 7.5 pmol mol ^−1^ for COS (Kooijmans et al., 2016), the Γ _COS_ value at 25 °C remains statistically robust.**

**Kooijmans LMJ, Uitslag NAM, Zahniser MS, et al.: Continuous and high-precision atmospheric concentration measurements of COS, CO2, CO and H2O using a quantum cascade laser spectrometer (QCLS). Atmos. Meas. Tech. 2016;9. 10.5194/amt-9-5293-2016 Insufficient attention was paid to the potential importance of the temperature-dependent OCS uptake from the empty cuvette.  For example, at 25C, the uptake of OCS varies from 0 to -12.5.  This is a big range and obviously highly significant when considering the question of compensation points and very low fluxes at low [OCS] concentrations. When I see in Figure 4 that the uncertainty of the flux is about 5 pmol m-2 s-1 at 100 pmol mol-1, this seems discordant with such a potentially large range in fluxes from the empty chamber. I acknowledge that this may be dealt with in the statistics so I am not, per se, questioning the result but just asking for more careful deliberation about the potential impacts of the empty chamber flux. We appreciate the reviewer’s insightful comment highlighting the potential influence of temperature-dependent COS uptake from the empty cuvette. We agree that variations in background COS flux could affect the estimation of low leaf fluxes and compensation points, particularly under low COS mole fraction conditions. In the revised manuscript, we have clarified how this issue was quantitatively addressed. Specifically, we incorporated the residual variability of the empty chamber into the uncertainty analysis by propagating the RMSEs of 6.9 pmol mol⁻¹ for [COS]ₐ and 2.73 pmol m⁻² s⁻¹ for F _COS_ into the regression-derived Γ _COS_. This approach effectively accounts for the potential influence of empty-cuvette temperature dependence on the estimated compensation point. Furthermore, as shown in Section 3.1.1, we explicitly report that the Γ _COS_ value at 19.8 °C is statistically indistinguishable from zero when this propagated uncertainty is considered, while the Γ _COS_ at 25 °C remains significantly positive. This result indicates that, although some temperature-dependent background variability may remain, it does not alter the overall conclusion that Γ _COS_ increases with temperature. We have now added a short statement in Section 3.1.1 to explicitly acknowledge this potential influence and clarify that it was incorporated into our total uncertainty estimation.**

****<** **3.1.1. COS Compensation Point (Experiment 1** **)>****

**We quantified the regression-based Γ _COS_ using a linear function at two temperatures: Γ _COS_ was 55.0 ± 53.2 pmol mol ^−1^ at 19.8 °C and 138.7 ± 26.1 pmol mol ^−1^ at 25.0 °C (error estimates denote 95 % confidence interval). These results indicate that Γ _COS_ increases with rising T _leaf_, suggesting a potential temperature dependence. Although the empty-chamber regression effectively removed the baseline COS emission, some residual variability likely remained (RMSE = 6.9 pmol mol ^−1^ for [COS] _a_ and 2.73 pmol m ^−2^ s ^−1^ for F _COS_). To evaluate the potential influence of this unresolved background variability, we propagated these errors to the regression-derived Γ _COS_. When the uncertainty is included, the 95% confidence interval of Γ _COS_ widened from ± 53.2 to ± 93.4 pmol mol ^−1^ at 19.8 °C and from ± 26.1 to ± 48.9 pmol mol ^−1^ at 25.0 °C. The large uncertainty at 19.8 °C indicates that the corresponding Γ _COS_ value is statistically indistinguishable from zero. By contrast, the Γ _COS_ measured at 25 °C remains significant even when this propagated and instrumental uncertainty is considered. Even accounting for the reported QCLS uncertainty of approximately 7.5 pmol mol ^−1^ for COS (Kooijmans et al., 2016), the Γ _COS_ value at 25 °C remains statistically robust.   The authors acknowledge this in the Discussion but I also found myself concerned by the fact that the experiments were optimized using observations conducted under conditions where [OCS] was an order of magnitude higher than the compensation point yet these simulations were used to study compensation point dynamics. I understand the operational reasons for this and I think the authors were transparent about this but it leaves quite a lot of room for future studies. We agree that the optimization was performed under high COS mole fractions, which may limit the interpretation of compensation point dynamics under near-ambient concentrations. Our COS model indeed parameterizes *Γ _COS_* as a temperature-dependent function and does not explicitly include a diffusivity or the impact of inflowing COS mole fractions due to the limited stability of COS detection at low ambient levels. We have now clarified this limitation in the Discussion, noting that current measurement systems allow reliable detection only under elevated COS levels. We also emphasize that future work with improved precision at low COS concentrations will help determine whether *Γ _COS_* varies with ambient [COS] due to retro-diffusion or other physiological mechanisms.**

****<4.1.** **Uncertainties in measurements and models>** In addition to the experimental limitations discussed above, a practical limitation arises from the mole fraction range used for model optimization. Model parameters were optimized using data obtained under relatively high COS mole fraction conditions (~1000 pmol mol⁻¹) to ensure stable and precise flux measurements, whereas *Γ _COS_* was determined from experiments conducted at lower inflowing concentrations (100–600 pmol mol⁻¹). This discrepancy in concentration ranges may introduce some uncertainty when extrapolating parameters between optimization and compensation-point estimation. Nevertheless, the model formulation remains internally consistent, and the derived parameters are valid for interpreting the relative temperature-dependent behavior of COS exchange. Future experiments targeting near-ambient COS mole fraction levels will be valuable to further validate the applicability of these optimized parameters and to evaluate potential retro-diffusion effects under more natural conditions. < **4** **.2. Possible causes of a COS compensation point** **>** Taken together, the existence of *Γ _COS_* and its increase at higher temperatures may result from a combination of the factors discussed above. However, our current formulation of *Γ _COS_* is expressed solely as a temperature-dependent function and does not explicitly include retro-diffusion effects. As a result, while the observed increase in *Γ _COS_* with temperature is consistent with thermally driven processes, the potential contribution of internal diffusion feedbacks—particularly under elevated COS mole fractions—cannot be fully excluded. At present, reliable measurements can only be obtained under relatively high COS mole fractions, which limits our ability to fully constrain *Γ _COS_* dynamics near ambient conditions. Improving detection precision at low COS concentrations and incorporating explicit representations of internal diffusion in future models would enable a more direct evaluation of whether *Γ _COS_* variability originates from retro-diffusion or from physiological and biochemical processes. Moreover, extending such measurements to different phenological stages and species, and exploring interactions with microbial associations and environmental stressors, will be essential for developing a more mechanistic understanding of COS exchange in plants.   No page numbers.**

**Line 1: “quantifies the _____ gross CO2….”  Should that “largest gross”? We have modified the sentence to “Gross primary productivity (GPP) quantifies the largest terrestrial CO _2_ uptake flux in the global carbon cycle.”   Top  of Page 5: “stomatal closure like the case” We have revised the sentence to improve clarity. The phrase “like is the case for CO₂” has been replaced with “as is the case for CO₂” to ensure grammatical correctness and a smoother flow.   Page 10: The text following Equation 7 needs to be rewritten We appreciate the reviewer’s suggestion. The sentence has been revised for clarity. It now reads: <2.2.1. General concept> Although accounting for the H _2_O ternary system is important, its influence on the calculated mole fractions was found to be negligible. The smaller ternary effects within the leaf boundary layer were therefore not included, as they scale with E / g _bw_ (von Caemmerer & Farquhar, 1981, Equation B10) and are expected to be negligible under our experimental conditions (large g _bw_ and small E). Note that the mole fractions calculated by the model are expressed on a wet-air basis, whereas the observed mole fractions are reported on a dry-air basis. The moisture removal applied to model outputs is described at the end of this section. To test Hypothesis 2, we analyzed how the intercellular mole fraction [gas] _i_ influences the net flux. The flux was computed using Equation 3, Equation 6, and Equation 7 to determine [gas] _i_, and then applied to the flux-gradient relationship:**

# Reviewer report: Reviewer response for version 1

**DOI:** 10.21956/openreseurope.21895.r58706

**Published:** 2025-09-23

**Authors:** Jerome Ogee

**Affiliations:** 1 ISPA, Institut National de Recherche pour l'Agriculture l'Alimentation et l'Environnement Centre Nouvelle-Aquitaine Bordeaux (Ringgold ID: 113907), Villenave-d'Ornon, Aquitaine-Limousin-Poitou-Charentes, France; 2 Bordeaux Sciences Agro, Villenave d’Ornon, France

The manuscript presents original gas exchange data of COS and CO2 fluxes on sunflower leaves that indicate COS emissions from leaves that increase with temperature. This is a rather unique dataset because very few groups have managed to measure leaf COS gas exchange due to the presence of COS-emitting surfaces in such systems that complicate the interpretation of the results (and the identification of COS emissions from the leaf itself). The authors seem to have addressed this issue rather well, and the data seems of good quality, and in support of what is advanced in the title and abstract.

 Despite all these very good points, writing the review was quite difficult because I found some issues in the manuscript that needed to be addressed and wanted to propose the most constructive way of doing so. I think I came up with a good plan that I hope the authors will agree with and follow. In particular, I did not find the modelling aspect of the study very convincing, as many assumptions and shortcuts were not well justified (see below). More importantly, I think that the modelling exercise is not needed and weakens the study (because of those many assumptions and shortcuts) and therefore I strongly suggest to refocus the paper strictly on the data, which is sufficient to support the main findings (or test the three main hypotheses). By doing so, the authors will need to revise their data analysis as well, because the equations to compute the gas exchange fluxes and conductances seem to contain some mistakes (in the text at least, I have not checked the code), leaving the reader with the impression that the theory behind has been misunderstood or at least wrongly applied. This is a concern because the data seems of good quality and there are good chances that the results may not change much after corrections, but we can never be sure until those changes are made. I think by refocusing the paper on the data analysis only would make the manuscript much shorter and stronger, with a clearer message. The modelling part could be the topic of another (separate) study, but this would require a better justification of some modelling assumptions beforehand. Below I have listed my major concerns and provide directions to address them.

 Because the manuscript has no line numbers, I refer below to equation or section numbers instead of line numbers.

 In Eq 1 the authors do not account for the change of air flow on the outlet of the cuvette due to the extra moisture brought about by transpiration (see Appendix 2 of von Caemmerer and Farquhar 1981 - noted vCF81 from now - or the LICOR’s manual). These equations also do not account for the fact that, unlike the LICOR’s IRGA, the QCLS instrument measures CO2 (and COS) mixing ratios on a dry air basis. Note also that a different equation should be used to estimate transpiration (see Eq B5 in vCF81).

 In Eqs 3-5 and 6-7, the air volume in the storage term should not be the entire chamber volume, and also because the ternary correction terms are not accounted for properly. Also, in Eq 3, the flux calculation should account for the difference in mass flow entering and exiting the leaf cuvette (see comment about Eq 1) and the "flux-gradient" calculation should include ternary corrections. Eq 6 implicitly assumes that the ternary correction is only important within the stomata, and not in the leaf boundary layer, and Eqs 6-7 are inconsistent with Eq 8 that neglects ternary correction. Fortunately, these "step-by-step" equations are not needed to interpret gas exchange data, and global equations like Eq B17 in vCF81 should be used instead (but with a clear distinction of mixing ratios on a dry or wet air basis).

 In Eq 8, ternary corrections are missing (see above and Eq B17 in vCF81).

 Eq 10 is correct but the distinction between mixing ratios on a dry and wet air should be made earlier. Also, the whole sentence leading to this Eq 10 is extremely confusing and should be rewritten and simplified.

 Eq 11 and the notion of “mesophyll conductance” for water vapour is quite debated (i.e. assuming that the air is not saturated in the leaf stomatal cavity) and unjustified here where the leaves were not exposed to very dry air or high temperature or light levels. Also the value of 10 mol/m2/s for that conductance is so high (4 times larger than the boundary-layer conductance) that its effect is probably negligible, and so introducing this new variable (and Eq 13 below) is pointless. I would recommend to neglect it, as it is normally done in the LiCOR manual.

 Eqs 14-17 are not needed to interpret gas exchange data, and test the three hypotheses, because only fluxes and LRU values are shown afterwards. Also in Eq 15, it should, not be CO2_i but CO2_c (at the chloroplast level).

 In Eq 18, why assuming that mesophyll conductance to COS is only biochemical? There should be a diffusional part considered. But again, this parameterisation of mesophyll conductance for COS is not needed for testing the three hypotheses and interpret gas exchange data.

 Eqs. 19-21 present three possible temperature responses for the estimated “compensation point" for COS. It is a bit strange to show results (Figure 3 with observational data points) here. I think this section (and Eq 19-21) should be brought in the Results section directly. It is not part of the Methods.

 Eqs 22 neglects changes in mass flow between the inlet and outlet (see Eq B7 in vCF81) and Eqs 23-24 are also incorrect (see comment above on Eq 6-7). I don’t understand why stomatal conductance is not derived directly from the measurements (see LiCOR’s manual or Eq B14-15 in vCF81). Given the high boundary-layer conductance (about 3-4 times stomatal conductance), the sensitivity of stomatal conductance to the exact choice of “K" is very small (see LICOR manual) and it is much more reliable than an “optimized” value based on a simplified model of photosynthesis (and COS uptake), with lots of unknown parameter values. Also, it seems odd to estimate a compensation point for COS using a model that requires a parameterisation of this compensation point. If the idea is to compute the COS concentration in the intercellular air space, an equation similar to Eq B18 In vCF81 could be used (see also Stimler et al 2010). But in the end, the compensation point for COS is only estimated from the intercept of the regression line of FCOS vs [COS]a (Figure 4) so only COS flux calculations are needed, and sections 2.2.3, 2.2.4 and 2.3 could be removed from the manuscript.

 Section 2.4 could also be removed if the modelling part is taken out (as I suggest). There is no need of a biochemical model that needs to be “parameterised” to discuss the gas exchange results presented here. As I explained above, what needs to be done instead is to compute gas exchange parameters (fluxes, conductances, etc) following the LICOR manual (but accounting for the fact that the QCLS computes mixing ratios on this dry air basis). This would make the manuscript much shorter and easier to read and follow.

 The discussion about measurement errors on stomatal conductance at the end of section 2.4.1 lacks some justification (where does the extra measurement error comes from?). But this part should be removed anyway, because this measurement error was only used for the biochemical model optimization that I propose to get rid of.

 In section 2.4.2 and Eq 25, the introduction of “weights" in the cost function is difficult to understand because it seems that those weights are there to wipe out differences in observational error between CO2, COS and H2O. But the cost function is normalised by these error terms for a reason, no? And again if the biochemical model optimization is removed, section 2.4 is also removed, and this comment is not relevant anymore.

 In section 3.1.1, you mention a “20% increase" of what? Stomatal conductance? Also it would be good to propagate errors due to the COS emission from the chamber (the scatter on Fig 1 is rather large) and clearly state whether the retrieved composition point are all statistically different from zero.

 I have not finalized the review of the end of the manuscript but I see that Figs 7-9 and most Tables except Table 1 could be removed as they show results from the biochemical model optimization that, to me, should be removed because I do not see what it brings to the study. My suggestions would make the paper much more digest and strong, and the main message should stay almost unchanged. I hope the authors will agree with this suggestion.

Is the study design appropriate and does the work have academic merit?

Yes

Is the work clearly and accurately presented and does it cite the current literature?

Partly

If applicable, is the statistical analysis and its interpretation appropriate?

Partly

Are all the source data underlying the results available to ensure full reproducibility?

Yes

Are the conclusions drawn adequately supported by the results?

Partly

Are sufficient details of methods and analysis provided to allow replication by others?

Partly

Reviewer Expertise:

Land-atmosphere interactions, micrometeorology, plant ecophysiology, carbon cycle

I confirm that I have read this submission and believe that I have an appropriate level of expertise to confirm that it is of an acceptable scientific standard, however I have significant reservations, as outlined above.

# Response

**Published:** 2025-12-03

**Authors:** Ara Cho

**Affiliations:** Meteorology and Air Quality, Wageningen University & Research, Wageningen, Gelderland, The Netherlands

In Eq 1 the authors do not account for the change of air flow on the outlet of the cuvette due to the extra moisture brought about by transpiration (see Appendix 2 of von Caemmerer and Farquhar 1981 - noted vCF81 from now - or the LICOR’s manual). These equations also do not account for the fact that, unlike the LICOR’s IRGA, the QCLS instrument measures CO2 (and COS) mixing ratios on a dry air basis. Note also that a different equation should be used to estimate transpiration (see Eq B5 in vCF81).

 We thank the reviewer for pointing out this important issue regarding outlet air flow and the mixing ratio basis. We note that this effect was initially considered during model development; however, implementing a full correction would require the outflowing H _2_O mole fraction—estimated by the model—to be fed back as an input variable to adjust the outlet flow, which is beyond the current model framework. Therefore, we chose not to apply this correction explicitly but verified that its effect is minor.

 In the revised manuscript, we have stated that our analysis is based on dry-air mixing ratios, consistent with the QCLS instrument, while the model internally uses wet-air mixing ratios. The conversion between these two bases is explicitly introduced in Equation 10. Regarding the outlet mass flow, we have now added an explanation and citation to von Caemmerer & Farquhar (1981, Appendix 2, Eqs.B3-B5), which describe the correction for increased outlet flow due to transpired water vapor. Under our experimental conditions ([H2O]_out = 15–30 mmol mol⁻¹), this correction leads to a change in calculated fluxes of ≤ 3%, which we consider negligible for the conceptual interpretation of CO₂, COS, and H₂O fluxes. Importantly, this correction was not applied to either the observed or modeled fluxes, so the model–observation comparison remains internally consistent. We have added a statement in the Methods acknowledging this assumption and its potential effect (<3%).

 Here, the [gas]_in and [gas]_out represent the mole fractions of COS (pmol mol *^−^* ^1^) and CO _2_ (µmol mol *^−^* ^1^) in the air entering and exiting the chamber, which are measured by the QCLS on a dry-air basis. The leaf area *S* (m ^2^) was 9 cm ^2^. Although transpiration slightly increases the outlet air flow due to the addition of water vapor (von Caemmerer & Farquhar, 1981, Appendix 2), the resulting increase in total molar flow (< 3% under [H₂O]_out = 15–30 mmol mol⁻¹) was neglected in Equation 1 for simplicity. Since this was only a small effect, it was not considered in the flux calculations and was consistently omitted throughout the model analysis. The CO _2_ leaf assimilation rates obtained from the QCLS measurements closely matched those from the LI-6800 ( *R *= 0.96, mean difference = 0.18 µmol m *^−^* ^2^ s *^−^* ^1^), confirming the accuracy of the CO _2_ flux measurements.  

 In Eqs 3-5 and 6-7, the air volume in the storage term should not be the entire chamber volume, and also because the ternary correction terms are not accounted for properly. Also, in Eq 3, the flux calculation should account for the difference in mass flow entering and exiting the leaf cuvette (see comment about Eq 1) and the "flux-gradient" calculation should include ternary corrections. Eq 6 implicitly assumes that the ternary correction is only important within the stomata, and not in the leaf boundary layer, and Eqs 6-7 are inconsistent with Eq 8 that neglects ternary correction. Fortunately, these "step-by-step" equations are not needed to interpret gas exchange data, and global equations like Eq B17 in vCF81 should be used instead (but with a clear distinction of mixing ratios on a dry or wet air basis).

 We thank the reviewer for the insightful comments. We agree that the air volume in the storage term should not correspond to the entire chamber volume. In our formulation (Eqs.3-5), the first term on the left-hand side represents the rate of gas accumulation (d[gas]/dt), but under steady-state conditions, this term becomes negligible. Accordingly, we have clarified in the revised manuscript that V_CV refers to the effective air volume representing the gas-exchange region near the leaf surface, rather than the total chamber volume.

 We note that the ternary corrections were already included in our Eqs. 6-7 following Jarman (1974) and von Caemmerer and Farquhar (1981) (vCF81). These equations account for the coupling between H _2_O diffusion and COS & CO _2_ transport at the stomatal level through the “F_H2O([gas]_b+[gas]_i)/2” term, which is formulated on a wet-air mole fraction basis consistent with the diffusion formulation in vCF81.

 By contrast, the QCLS instrument measures COS and CO _2_ mole fractions on a dry-air basis. To ensure consistency, the model outputs were converted from wet-air to dry-air mole fractions using the relation described in Equation 10 before comparison with observations. We have clarified this distinction in the revised manuscript.

 Equation 8, by contrast, is a simplified steady-state expression used only for diagnostic evaluation (i.e., to compute AFR in Section 3.2.3), not for model calculation. Therefore, although Equations 6–8 are not fully consistent, the ternary system at the stomatal level is appropriately represented within the model.

 For the boundary layer, von Caemmerer & Farquhar (1981, Equation B10) showed that the ternary correction scales as c¯E/gbc¯E/gb, where \bar{c} is the mean gas mole fraction between ambient and boundary air. Although this term cannot be precisely quantified in our dataset (as \bar{c} was not directly measured), the ternary correction at the boundary layer is expected to be negligible under our experimental conditions because g_b (~2.44 mol m⁻² s⁻¹) was much larger than g_s and transpiration (E) was small (0.0028–0.0081 mol m⁻² s⁻¹).

 Therefore, we applied ternary corrections only at the stomatal interface (Eqs. 6–7) and neglected them in the boundary layer for computational efficiency, where their effect is theoretically minor.

 Here, V_m (m ^3^ mol ^−1^) is the molar volume, and V_CV (m ^3^) is the effective air volume representing the gas-exchange region near the leaf surface. Under steady-state conditions, the first term on the left-hand side of Equation 3, Equation 4, and Equation 5, which represents the rate of gas accumulation, becomes negligible. To account for the different properties of COS and CO _2_ to H _2_O, the stomatal conductances ( g_s,COS and g_s,CO2) and the boundary layer conductances ( g_b,COS and g_b,CO2) are scaled proportionally to the conductance of water vapor (g _sw_ and g _bw_) …

 For the COS and CO _2_ exchange at the stomatal level, a ternary system with H _2_O and air should be considered, because the transpiration ( *F*_H_2O (mol m *^−^* ^2^ s *^−^* ^1^)) is significantly larger than COS and CO _2_ assimilation (Jarman, 1974; von Caemmerer and Farquhar, 1981). Thus, we added the ternary term in Eqs. 4 and 5 for the COS and CO _2_ models:

 …Equation 6 7…

 Although accounting for the H _2_O ternary system is important, its influence on the calculated mole fractions was found to be negligible. The smaller ternary effects within the leaf boundary layer were therefore not included, as they scale with E / g_bw (von Caemmerer & Farquhar, 1981, Equation B10) and are expected to be negligible under our experimental conditions (large g_bw and small E). Note that the mole fractions calculated by the model are expressed on a wet-air basis, whereas the observed mole fractions are reported on a dry-air basis. The moisture correction applied to model outputs is described at the end of this section. To test Hypothesis 2, we analyzed how the intercellular mole fraction [gas]_ _i_ influences the net flux. The flux was computed using Equation 3, Equation 6, and Equation 7 to determine [gas] __i_, and then applied to the flux-gradient relationship:  

 In Eq 8, ternary corrections are missing (see above and Eq B17 in vCF81).

 We thank the reviewer for pointing this out. We clarify in the text that Equation 8 is not part of the model optimization or flux calculation; rather, they are used solely to define the Apparent Flux Ratio (AFR) introduced later in Section 3.2.3. This distinction ensures conceptual consistency with the vCF81 framework while keeping the equations’ role in our analysis transparent.

 The term (1-[gas] _i_ [gas] _a_^-1^) represents the effect of changes between internal and ambient mole fractions on the gas conductance and deposition velocity, termed Ambient Fraction Remaining (AFR). This concept is utilized in Sect. 3.2.3 for interpreting Hypothesis 2. Strictly speaking, the mass flow difference between the inlet and outlet and the ternary effect should be considered in Equation 8 and Equation 9. However, these effects are expected to be minor under our experimental conditions (see above), and Equation 8 and Equation 9 were used only to examine the conceptual relationship between [gas] _i_ and flux. Therefore, for simplicity, these correction terms were omitted. Note that this omission does not affect the interpretation of AFR in Section 3.2.3.

****

 Eq 10 is correct but the distinction between mixing ratios on a dry and wet air should be made earlier. Also, the whole sentence leading to this Eq 10 is extremely confusing and should be rewritten and simplified. In the revised manuscript, the distinction between *dry-air* and *wet-air* mixing ratios is now introduced earlier in the Methods section, prior to Equation 10, to improve clarity. The paragraph leading to Equation 10 has also been rewritten to be more concise and straightforward, highlighting only the key point: the QCLS instrument reports CO₂ and COS as *dry-air* mixing ratios, whereas the gas exchange model operates with *wet-air* mole fractions. Accordingly, Equation 10 provides the conversion between the two definitions.

 <2.2.1. General concept>

 Our leaf conductance model simulates the concurrent exchange of COS, CO _2_, and H _2_O in a plant leaf with the conditions of the laboratory experiments. Since these gases share the same stomatal pathway, their simultaneous modeling helps us understand the mechanisms of leaf conductance. The model calculates mole fractions on a wet-air basis, while the measurements are reported on a dry-air basis. Accordingly, conversions between dry- and wet-air mole fractions were applied when comparing modeled and observed concentrations (see Section 2.1.1).  Additionally, the model assumes that gas exchange reaches an equilibrium that we tried to attain in the conducted experiments. All model variables that remain constant in the model are listed in Table 2. Variables that are excluded from Table 2 are targets for optimization as explained in Section 2.3.

 From experiments, we measured [ *gas*] *_out_*, while the leaf conductance model calculates [ *gas*] *_a_*. Because the chamber air was well mixed, the modeled [gas] _out,est_ is considered equivalent to [gas] _a_ for model-observation comparison. Thus, [gas] _out,est_ is used for [gas] _a_ to interpret experimental data. In the model, the estimated mole fractions for COS and CO _2_ are based on wet air, whereas the QCLS measures dry-air mole fractions. Therefore, to compare model results with observations, the modeled wet-air mole fractions were converted to their dry-air equivalents using the observed outgoing H _2_O mole fractions ([H _2_O] _out,obs_ (mmol mol ^−1^)):

 … Equation 10…

 where [H _2_O] _out,obs_ is the observed outgoing H _2_O mole fraction (mmol mol *^−^* ^1^).  For [COS] _in,obs_, which was measured after moisture removal, the same correction (Equation 10) was applied using [H _2_O] _in,obs_ instead of [H _2_O] _out,obs_ to convert from the dry-air to the wet-air basis before use in the conductance model.  

 Eq 11 and the notion of “mesophyll conductance” for water vapour is quite debated (i.e. assuming that the air is not saturated in the leaf stomatal cavity) and unjustified here where the leaves were not exposed to very dry air or high temperature or light levels. Also the value of 10 mol/m2/s for that conductance is so high (4 times larger than the boundary-layer conductance) that its effect is probably negligible, and so introducing this new variable (and Eq 13 below) is pointless. I would recommend to neglect it, as it is normally done in the LiCOR manual.

 We thank the reviewer for this comment and agree that the assumption of a non-saturated intercellular airspace (RH < 100%) is relevant only under very dry or extreme conditions, which were not studied in our experiments.

 To verify that this assumption does not affect our results, we retained a mesophyll conductance for water vapor (gₘ _,H₂O_) in the model as a *sanity check*. Testing a wide range of gₘ, _H₂O_ values (<10 mol m⁻² s⁻¹) confirmed that its effect on the computed fluxes and internal mole fractions is indeed negligible.

 We will clarify in the revised text that gₘ _,H₂O_ was used only to test the sensitivity to possible non-saturation in the intercellular airspace, and that it does not alter the modeled outcomes under our experimental conditions.

 In our experiments, we observed water evaporation from leaves. We aimed to model this evaporation at the mesophyll level and its subsequent transport out of the leaves, governed by *g_sw*. The unit of H _2_O mole fractions in all layers is mmol mol *^−^* ^1^, and the unit of the flux is mmol m *^−^* ^2^ s *^−^* ^1^. To represent the internal water vapor conditions, [H _2_O] _c_ was calculated assuming that water vapor within the mesophyll intercellular airspace is saturated. However, we allowed for relative humidity within the intercellular airspace (RH _i_ (%)) to be less than 100 %, based on previous studies (Cernusak *et al.*, 2018; Wong *et al.*, 2022). To achieve this, we introduced a mesophyll conductance for water vapor ( *g _mw_*) and set it to an arbitrary value of 10 mol m *^−^* ^2^ s *^−^* ^1^, approximately ten times the largest observed value of *g_sw*. This parameter was included to test the sensitivity of the model to possible non-saturation within the intercellular airspace. *The optimized g_sw also showed consistent behavior. Comparisons of the optimized mean g_sw values of 0.64 mol m ^−2^ s ^−1^ with the prior values of 0.62 mol m ^−2^ s ^−1^ reveal slight differences. Errors in g_sw were reduced from 0.09 to 0.05 mol m ^−2^ s ^−1^. The optimized model yielded an average relative humidity within the leaf (RH _i_) of 98.82 ± 0.04 %. These results confirm that allowing for slight non-saturation in the intercellular airspace (through gₘ _,H₂O_) had a negligible impact on the modeled fluxes, validating the assumption that the air within the intercellular airspace was effectively saturated under our experimental conditions. Simulations further confirmed that varying g _mw _(< 10 mol m⁻² s⁻¹) did not alter the modeled fluxes or mole fractions, indicating that its effect was negligible under our experimental conditions.*  

 Eqs 14-17 are not needed to interpret gas exchange data, and test the three hypotheses, because only fluxes and LRU values are shown afterwards. Also in Eq 15, it should, not be CO2_i but CO2_c (at the chloroplast level).

 We respectfully disagree that Equations14–17 are unnecessary. Although the main measurement outcomes presented in the Results are fluxes and LRU values, these equations are essential to interpret the underlying internal processes that directly relate to our three hypotheses (e.g., the temperature dependence of COS compensation point and the differential stomatal impacts on COS v.s. CO _2_). The layer-specific intercellular mole fractions derived from these equations provide mechanistic insight into gas diffusion and were thus retained for completeness.

 Regarding Equation 15, we agree that, in principle, it should be expressed as a function of the CO _2_ partial pressure at the chloroplast level (p[CO _2_] _c_), as RuBisCO carboxylation occurs within the chloroplast stroma. However, our model does not explicitly include mesophyll conductance (g _m,CO2_), and thus p[CO _2_] _c_ cannot be directly resolved. In such cases, it is standard practice to approximate p *C* *O* *2* *c* *≈* *p* *C* *O* *2* *i*  (Harley et al., 1992), assuming negligible mesophyll resistance.

 …Equation 25… where *p*[CO _2_] _i_ (Pa) and *p*[O _2_] _i_ (Pa) are the partial pressures of CO _2_ and O _2_ in the inter-cellular air space. *K _c_* (Pa) and *K _o_* (Pa) are Michaelis-Menten constants for CO _2_ and O _2_, respectively. *Γ* *^∗^* *_CO2_* **(Pa) stands for the CO _2_ compensation point independent of dark respiration. Strictly speaking, RuBisCO carboxylation occurs within the chloroplast stroma and should depend on the chloroplast CO _2_ partial pressure (p[CO _2_] _c_), rather than *p*[CO _2_] _i_. However, our model does not explicitly include mesophyll conductance (g _m,CO2_), and thus *p*[CO _2_] _c_ cannot be directly resolved. Accordingly, *p*[CO _2_] _i_ was used as a proxy for *p*[CO _2_] _c_, following the classical formulation of Farquhar et al. (1980).     

 In Eq 18, why assuming that mesophyll conductance to COS is only biochemical? There should be a diffusional part considered. But again, this parameterisation of mesophyll conductance for COS is not needed for testing the three hypotheses and interpret gas exchange data.

 We agree that, in principle, mesophyll conductance to COS ( *g* *m,COS* ) includes both diffusional and biochemical components. In the original text, we referred to the “biochemical” aspect because the overall conductance is strongly modulated by carbonic anhydrase (CA) activity, which catalyzes COS hydrolysis. In the revised manuscript, we clarify that *g* *m,COS*  conceptually represents the combined effect of diffusion through mesophyll tissues and biochemical conversion by CA. Because CA activity is very fast, the apparent temperature response of *g* *m,COS*  can be largely attributed to biochemical limitation, but we acknowledge that it may also reflect diffusional effects. We also note that the inclusion of *g* *m,COS*  was essential for evaluating the potential for COS emission (Hypothesis 1), as excluding it would prevent quantifying internal COS gradients and reversibility of net fluxes. We therefore retained Equation 18 but revised the surrounding text to clarify its conceptual basis and limited purpose.

 We constructed the COS model to estimate the gross COS flux rate ( *F _COS_*, pmol m *^−^* ^2^ s *^−^* ^1^) and mole fractions of COS in each layer (pmol mol *^−^* ^1^). Accurate modeling of COS leaf uptake requires representing mesophyll conductance, which integrates both the diffusional transport of COS and biochemical conversion by CA.

 Earlier approaches treated mesophyll diffusion and CA activity as linearly proportional to Rubisco’s Vmax (Berry et al., 2013). However, this simplification was shown to bias COS flux estimates, leading to the introduction of a revised CA-based temperature formulation (Cho et al., 2023). More recently, Lai et al. (2024) emphasized the importance of explicitly accounting for mesophyll diffusion in COS uptake models but also noted the lack of reliable parameterization for COS-specific mesophyll diffusion.

 Building on these previous findings, we modeled biochemical conductance of COS in the mesophyll (g _m,COS_ (mol m ^−2^ s ^−1^)) using the CA activity–based function proposed by Cho et al. (2023), which implicitly represents mesophyll diffusion while minimizing estimation bias through its revised temperature dependence. This function describes the temperature dependence of CA activity using a specified Arrhenius equation and Michaelis-Menten Kinetics (Cho et al., 2023; Daniel et al., 2010; Ogée et al., 2016; Sun et al., 2015):

 From a modeling perspective,  only the enzyme CA is considered in determining the mesophyll conductance of COS. However, other enzymes, such as RuBisCO and phosphoenolpyruvate carboxylase (PEP-C), are known to also catalyze COS uptake (Lorimer and Pierce, 1989; Protoschill-Krebs and Kesselmeier, 1992). The contribution of RuBisCO to COS uptake is relatively minor compared to CA (Protoschill-Krebs *et al.*, 1996), and its activity has not yet been quantified.

 An additional source of uncertainty arises from the simplified representation of COS mesophyll conductance in our model. While mesophyll diffusion was not explicitly parameterized, it was implicitly represented through the temperature-dependent CA activity function (Cho et al., 2023) that integrates both diffusional and biochemical processes. Nevertheless, this implicit treatment may not fully capture variations in diffusional resistance under different environmental or structural conditions, and thus its influence on the temperature dependence of COS uptake cannot be completely excluded. Future modeling studies incorporating explicit mesophyll diffusion parameterization—beyond the current CA-based formulation—would help to disentangle the relative contributions of diffusional and biochemical controls, as well as potential COS emissions from leaves, in regulating COS exchange.  

 Eqs. 19-21 present three possible temperature responses for the estimated “compensation point" for COS. It is a bit strange to show results (Figure 3 with observational data points) here. I think this section (and Eq 19-21) should be brought in the Results section directly. It is not part of the Methods.

 We appreciate the reviewer’s remark. We have carefully considered this suggestion. However, Figure 3 and Equations 19–21 are retained in the Methods section because they represent the initial parameterization and uncertainty characterization used for the subsequent model fitting. The figure illustrates the prior settings and error structures applied in the optimization framework, rather than observational or derived results.

 For clarity, we have slightly revised the caption and text to emphasize that these correspond to model initialization steps rather than final experimental outcomes. We believe this organization helps readers distinguish between methodological setup and empirical findings presented later in the Results section.

**(Revised manuscript – the red sentences were added)**

 Figure 3 shows these four Γ _COS_ functions (S1–S4) representing the prior temperature-dependent parameterizations tested in the modeling framework with their imposed uncertainties. The figure also shows the experimental Γ _COS_ estimates from Experiment 1, included solely for reference to illustrate the initial parameter alignment.

 Figure 3. Prior temperature functions of the COS compensation point (Γ _COS_) (lines) with their error ranges (shaded area). Blue triangles show the regression-derived Γ _COS_ from Experiment 1, included for reference to illustrate the initial parameter setup. The S1 model assumed Γ _COS_ = 0 pmol mol ^−1^. Model S2 describes Γ _COS_ as a linear relationship with T _leaf_ (a), while models S3 and S4 use an Arrhenius equation (b).  

 Eqs 22 neglects changes in mass flow between the inlet and outlet (see Eq B7 in vCF81) and Eqs 23-24 are also incorrect (see comment above on Eq 6-7).

 We thank the reviewer for this comment. In the revised manuscript, we have clarified the assumptions regarding outlet mass-flow and ternary corrections based on the reviewer’s earlier feedback. Specifically, we now state that although transpiration slightly increases the outlet air flow, the effect (<3%) was neglected to retain easy analytical expressions, consistent with the assumption in Equation 1. Likewise, the ternary effects at the boundary layer were considered negligible under our experimental conditions (large g_bw, small E), following Equations 6–7. These clarifications have been explicitly added after Equation 22 to ensure transparency. As these approximations were consistently applied throughout the analysis (both in observations and model calculations), they do not affect the internal consistency or interpretation of our results.

 Because the temperature-dependent behavior of Γ _COS_ in Experiment 1 was derived from a limited set of measurements under only a few temperatures, additional validation was required to test whether the modeled Γ _COS_ functions are consistent across broader environmental conditions.

 To support this validation, we incorporated the indirectly derived Γ _COS_ from 48 data points in Experiments 2 and 3.

 By calculating [COS] _c_ using our optimized model parameters in Equation 3, Equation 6, and Equation 7, we were able to indirectly estimate Γ _COS_ and compare it with the modeled Γ _COS_.

 First, we calculated [COS] _b_ from the measured COS mole fractions with the rearranged Equation 3: …Equation 3…  

 where [COS] _in_ and [COS] _a_, along with *AF *, *S*, and *g _b,COS_*, were applied from Experiments 2 and 3. Due to thorough mixing within the cuvette, we assumed [COS] _out_ to be equivalent to [COS] _a_ and used it accordingly (refer to Sect. 2.2).

 Although transpiration slightly increases the outlet air flow, this effect (< 3%) was neglected to simplify the analytical expressions, consistent with the assumptions described in Equation 1. This formulation follows the same assumptions as in Equation 3 regarding ternary effects at the boundary layer, which were found to be negligible under our experimental conditions. Here, [COS] _in_ and [COS] _out_ were converted from a dry air basis to a wet air basis. We then used the derived [COS] _b_ to calculate [COS] _i_ using the rearranged Equation 6:    

 I don’t understand why stomatal conductance is not derived directly from the measurements (see LiCOR’s manual or Eq B14-15 in vCF81). Given the high boundary-layer conductance (about 3-4 times stomatal conductance), the sensitivity of stomatal conductance to the exact choice of “K" is very small (see LICOR manual) and it is much more reliable than an “optimized” value based on a simplified model of photosynthesis (and COS uptake), with lots of unknown parameter values.

 We appreciate the reviewer’s comment and agree that the LI-6800 provides reliable estimates of stomatal conductance (g_sw) based on water vapor exchange. However, in our model, we want to test whether the g_sw values from the LI-6800 (we start our optimization from these values) are consistent with the observed exchange CO₂ and COS. When adjustments within a prescribed error range are profitable for better simulations of CO _2_ and COS, we allow for such adjustments in our optimization framework. This approach allows for a physically consistent representation across species and may compensate for potential biases associated with measurement uncertainties (e.g., the K parameter and cuvette humidity corrections). To our knowledge, this novel aspect in the interpretation of multi-species chamber observations has never been tested before. We therefore are keen to keep this new modelling framework in the paper, but agree with the reviewer that the model should not introduce biases by making wrong assumptions.

 A corresponding further clarification has been added in the Methods section.

 The LI-6800 derives *g_sw* from water vapor exchange with its own uncertainties. Because gₛ _w_ similarly affects the exchange of H₂O, CO₂, and COS through the same shared stomatal pathway, we optimize gₛ _w_ in a coupled framework using flux information from all three gases to ensure internal consistency across species. The novelty of this framework lies in explicitly coupling these fluxes to derive a more physically consistent constraint on g_sw, while also addressing potential uncertainties in g_sw derived from LI-6800 measurements (e.g., stomatal ratio parameter K; Sect. 2.1.1).

 Initial values for *g_sw *were taken from the LI-6800 measurements during Experiments 2 and 3. We applied a random error of 0.08 mol m *^−^* ^2^ s *^−^* ^1^, representing the standard deviation of *g_sw *from Experiment 3, despite efforts to maintain a constant *g_sw *( *± *0.06 mol m *^−^* ^2^ s *^−^* ^1^). In addition, we considered an extra measurement uncertainty of 0.02 mol m ^−2^ s ^−1^, based on known sources of instrumental bias—such as the leaf temperature underestimation (Garen et al., 2022) and background total leaf conductance to water observed in empty chambers (Hussain et al., 2024). We also added individual [H _2_O] _out,obs_ errors by normalizing [H _2_O] _out,obs_. The averaged initial *g_sw *value and its standard deviation (prior error) are 0.61 mol m *^−^* ^2^ s *^−^* ^1^ and 0.09 mol m *^−^* ^2^ s *^−^* ^1^, respectively.  

 Also, it seems odd to estimate a compensation point for COS using a model that requires a parameterisation of this compensation point. If the idea is to compute the COS concentration in the intercellular air space, an equation similar to Eq B18 In vCF81 could be used (see also Stimler et al 2010). But in the end, the compensation point for COS is only estimated from the intercept of the regression line of FCOS vs [COS]a (Figure 4) so only COS flux calculations are needed, and sections 2.2.3, 2.2.4 and 2.3 could be removed from the manuscript.

 We respectfully disagree. While it is true that the apparent COS compensation point can be empirically derived from the intercept of the F _COS_-[COS] _a_ relationship (as in Fig. 4), our study aims to go beyond this empirical regression and checks whether the model-data mismatches are smaller in models that include a compensation point formulation. The main objective of this work, as clearly stated in the Introduction, is to test three mechanistic hypotheses on the controls of COS exchange within leaves regarding (i) the role of stomatal and mesophyll conductances, (ii) the biochemical conversion by carbonic anhydrase, and (iii) the temperature dependence of these processes.

 To evaluate these hypotheses, the model must explicitly resolve the diffusion pathway and reaction kinetics from ambient air to the chloroplast. Therefore, estimating COS mole fractions in the chloroplast [COS] _c_ through the full conductance chain is essential. This approach allows us to assess how internal parameters influence the observed compensation behavior and to quantify their relative sensitivities.  

 Section 2.4 could also be removed if the modelling part is taken out (as I suggest). There is no need of a biochemical model that needs to be “parameterised” to discuss the gas exchange results presented here. As I explained above, what needs to be done instead is to compute gas exchange parameters (fluxes, conductances, etc) following the LICOR manual (but accounting for the fact that the QCLS computes mixing ratios on this dry air basis). This would make the manuscript much shorter and easier to read and follow.

 We appreciate the reviewer’s suggestion and fully agree that the observational dataset itself is of high quality and allows for a clear interpretation of COS and CO₂ exchange. Indeed, the measurements remain the central basis of our analysis.

 However, the inclusion of the modelling framework (Section 2.4) is essential for the specific objectives of this study. As stated in the Introduction, our goal is not only to describe observed fluxes but to test three mechanistic hypotheses concerning the internal regulation of COS exchange: (i) temperature-dependent Γ _COS_, (ii) stomatal conductance-dependent to internal exchange, and (iii) enzyme-specific temperature responses of CA and RuBisCO. These internal processes cannot be inferred directly from the measured fluxes, which is why a simple empirical treatment would not allow hypothesis testing. The model is therefore not a replacement for empirical analysis, but a diagnostic complement that integrates CO₂, COS, and H₂O exchange into a consistent mechanistic framework.

 Finally, we repeat that this represents the first attempt to jointly optimize gas-exchange parameters across all three gas species, allowing an independent and mechanistically consistent estimation of the COS compensation point — one of the central findings of this study.

 From a modeling perspective, only the enzyme CA is considered in determining the mesophyll conductance of COS. However, other enzymes, such as RuBisCO and phosphoenolpyruvate carboxylase (PEP-C), are known to also catalyze COS uptake (Lorimer and Pierce, 1989; Protoschill-Krebs and Kesselmeier, 1992). The contribution of RuBisCO to COS uptake is relatively minor compared to CA (Protoschill-Krebs et al., 1996), and its activity has not yet been quantified.

 An additional source of uncertainty arises from the simplified representation of COS mesophyll conductance in our model. While mesophyll diffusion was not explicitly parameterized, it was implicitly represented through the temperature-dependent CA activity function (Cho et al., 2023) that integrates both diffusional and biochemical processes. Nevertheless, this implicit treatment may not fully capture variations in diffusional resistance under different environmental or structural conditions, and thus its influence on the temperature dependence of COS uptake cannot be completely excluded. Future modeling studies incorporating explicit mesophyll diffusion parameterization—beyond the current CA-based formulation—would help to disentangle the relative contributions of diffusional and biochemical controls, as well as potential COS emissions from leaves, in regulating COS exchange.

 Beyond these structural uncertainties in the model, it is important to note that the conductance model was designed as an exploratory diagnostic tool. Its purpose was to integrate the coupled behavior of CO _2_, COS, and H _2_O exchange and to mechanistically interpret the observed fluxes. Despite its simplifications in not considering minor features, such as outlet mass flow change and boundary ternary effect, this framework represents the first attempt to jointly optimize gas exchange for the three gases, thereby providing a process-based means to independently estimate the COS compensation point. Overall, this approach highlights the potential of combined gas-exchange measurement and modeling to advance our understanding of COS biogeochemistry.

 The discussion about measurement errors on stomatal conductance at the end of section 2.4.1 lacks some justification (where does the extra measurement error comes from?). But this part should be removed anyway, because this measurement error was only used for the biochemical model optimization that I propose to get rid of.

 While we acknowledge that stomatal conductance (g_sw) can be derived directly following the LI-6800 manual, we retained its optimization for the reasons outlined above. The optimization ensures internal consistency across all fluxes rather than relying solely on water-vapor–based conductance values.

 Regarding the additional measurement uncertainty, we have now clarified its empirical basis in the revised text. The extra error term (0.02 mol m⁻² s⁻¹) was introduced conservatively to reflect known sources of uncertainty in LI-6800 gas-exchange measurements. Specifically, Garen et al. (2022) reported that the LI-6800 can underestimate leaf temperature, which propagates into water vapor pressure and hence g_sw estimation. In addition, Hussain et al. (2024) identified a nonzero total leaf conductance to water in empty-chamber tests, suggesting small but systematic instrument offsets. These findings justify the inclusion of a measurement uncertainty term in our optimization framework.

 The text in Section 2.4.1 has been revised accordingly to include these references and to emphasize that the error term was not introduced to inflate uncertainty but rather to conservatively account for known measurement limitations while maintaining robustness in the coupled CO₂–COS–H₂O optimization.

 Hussain SB, Stinziano J, Pierre MO, et al.: Accurate photosynthetic parameter estimation at low stomatal conductance: effects of cuticular conductance and instrumental noise. Photosynth. Res. 2024;160:111–124. 10.1007/s11120-024-01092-8

 Garen JC, Branch HA, Borrego I, et al.: Gas exchange analysers exhibit large measurement error driven by internal thermal gradients. New Phytol. 2022;236:369-384. 10.1111/nph.18347.

 <2.4. Parameter optimization> …

 The LI-6800 derives g_sw from water vapor exchange with its own uncertainties. Because g_sw similarly affects the exchange of H₂O, CO₂, and COS through the same shared stomatal pathway, we optimize g_sw in a coupled framework using flux information from all three gases to ensure internal consistency across species. The novelty of this framework lies in explicitly coupling these fluxes to derive a more physically consistent constraint on g_sw, while also addressing potential uncertainties in g_sw derived from LI-6800 measurements (e.g., stomatal ratio parameter K; Sect. 2.1.1).

 Initial values for *g_sw *were taken from the LI-6800 measurements during Experiments 2 and 3. We applied a random error of 0.08 mol m *^−^* ^2^ s *^−^* ^1^, representing the standard deviation of *g_sw *from Experiment 3, despite efforts to maintain a constant *g_sw *( *± *0.06 mol m *^−^* ^2^ s *^−^* ^1^). In addition, we considered an extra measurement uncertainty of 0.02 mol m ^−2^ s ^−1^, based on known sources of instrumental bias—such as the leaf temperature underestimation (Garen et al., 2022) and background total leaf conductance to water observed in empty chambers (Hussain et al., 2024). We also added individual [H _2_O] _out,obs_ errors by normalizing [H _2_O] _out,obs_. The averaged initial *g_sw *value and its standard deviation (prior error) are 0.61 mol m *^−^* ^2^ s *^−^* ^1^ and 0.09 mol m *^−^* ^2^ s *^−^* ^1^, respectively.  

 In section 2.4.2 and Eq 25, the introduction of “weights" in the cost function is difficult to understand because it seems that those weights are there to wipe out differences in observational error between CO2, COS and H2O. But the cost function is normalised by these error terms for a reason, no? And again if the biochemical model optimization is removed, section 2.4 is also removed, and this comment is not relevant anymore.

 We agree that the observational errors ( *σ* *obs* ) already normalize each flux term in the cost function. However, in our framework, the additional weighting factors (w _CO2_, w _COS_, w _H2O_) serve a different purpose. They are not intended to correct for measurement errors, but rather to balance the relative influence of each gas in the multi-objective optimization. We acknowledge that the final choices are rather subjective, but we have been very careful not to over-emphasize the conclusions based on the exploratory optimization process.

 Specifically, the magnitudes and temporal variabilities of CO₂, COS, and H₂O fluxes differ by more than an order of magnitude, even after normalization by their observational uncertainties. As we explain in the text, without weighting the optimization would be dominated by the CO₂ term, leading to suboptimal performance for COS and H₂O. The weights therefore ensure that all three gases contribute comparably to the minimization of the total cost function.

 Section 2.4.2 has been retained and revised for clarity. The introductory text now explicitly states that (i) observational errors are used for normalization, and (ii) weights are applied to achieve balanced optimization across gases with distinct flux magnitudes. We also emphasize that this coupled optimization framework is one of the novel aspects of our study, providing a unified treatment of CO₂–COS–H₂O exchange processes.  

 … Equation 25…

 The first term of the cost function penalizes deviations of the state *x *from the prior state vector *x _a_*. This penalty depends on *σ*, which represents the prior error in the state vector. This background term is introduced to keep state variables within reasonable boundaries (Brasseur and Jacob, 2017). The last three terms of the cost function calculate the costs associated with deviations between modeled ([ *gas*] *_out,est_*) and observed mole fractions ([ *gas*] *_out,obs_*) for COS, CO _2_, and H _2_O, respectively. Each term is normalized by its observational uncertainty to ensure consistent treatment of measurement errors, and each is scaled by optimization weights (W _bg_, W _COS_, W _CO2_, and W _H2O_) to balance the relative influence of each gas. These weights do not modify the observational errors but instead prevent any single gas—particularly CO _2_—from dominating the total cost. The determination of these weights is described in the following section.

 In section 3.1.1, you mention a “20% increase" of what? Stomatal conductance? Also it would be good to propagate errors due to the COS emission from the chamber (the scatter on Fig 1 is rather large)

 We thank the reviewer for this constructive comment. We have clarified in the revised manuscript that the reported 20 % increase refers specifically to stomatal conductance (g_sw), which increased from 0.76 to 0.90 mol m⁻² s⁻¹ during the temperature adjustment at higher T _leaf_. This clarification is now included in Section 3.1.1.

 Regarding the uncertainty propagation, we agree that the empty-chamber correction may not completely remove background variability. To assess its influence, we incorporated the residual RMSEs from the empty-chamber correction—6.9 pmol mol⁻¹ for [COS]ₐ (x-axis) and 2.73 pmol m⁻² s⁻¹ for F _COS_ (y-axis)—into the total uncertainty of the regression-derived Γ _COS_. Both sources of uncertainty were propagated to account for their combined impact on the regression intercept and, consequently, on the estimated compensation point.

 Specifically, we calculated the total propagated uncertainty as: \sigma_{\text{total}} = \sqrt{\sigma_{\text{regression}}^{2} + \sigma_{\text{chamber}}^{2}} where σ _regression_ is the standard error from the linear fit of F _COS_ versus [COS]ₐ, and σ _chamber_ combines the residual uncertainties from both F _COS_ and [COS]ₐ after the empty-chamber correction. This additional uncertainty was then included in the 95 % confidence interval of Γ _COS_. As a result, the 95 % confidence intervals of Γ _COS_ broaden from ±53.2 to ±93.4 pmol mol⁻¹ at 19.8 °C and from ±26.1 to ±48.9 pmol mol⁻¹ at 25.0 °C. The estimate at 19.8 °C includes zero, indicating that it is not statistically different from zero, whereas the estimate at 25.0 °C remains significantly positive, confirming a temperature-dependent increase in Γ _COS _and clearly states whether the retrieved composition points are all statistically different from zero.

 3.1.1. COS Compensation Point (Experiment 1)

 Figure 4 presents the results from Experiment 1 with Sunflower 1, aimed at determining *Γ _COS_* by measuring *F _COS_* while controlling [COS] _out_ and maintaining constant *g_sw *and *T _leaf_*. Minor fluctuations in *g_sw *occurred during temperature adjustments, increasing from 0.76 to 0.90 mol m ^-2^ s ^-1^ at higher temperatures. *F _COS_* shows a linear increase with increasing [COS] _out_, in agreement with findings from earlier studies (Gimeno *et al.*, 2017; Kesselmeier and Merk, 1993; Stimler *et al.*, 2010). We quantified the regression-based *Γ _COS_* using a linear function at two temperatures: *Γ _COS_* was 55.0 *± *53.2 pmol mol *^−^* ^1^ at 19.8 ℃ and 138.7 *± *26.1 pmol mol *^−^* ^1^ at 25.0 ℃ (error estimates denote 95 % confidence interval). These results indicate that *Γ _COS_* increases with rising *T _leaf_*, suggesting a potential temperature dependence.

 Although the empty-chamber regression effectively removed the baseline COS emission, some residual variability likely remained (RMSE = 6.9 pmol mol ^−1^ for [COS] _a_ and 2.73 pmol m ^−2^ s ^−1^ for F _COS_). To evaluate the potential influence of this unresolved background variability, we propagated these errors to the regression-derived Γ _COS_. When the uncertainty is included, the 95% confidence interval of Γ _COS_ widened from ± 53.2 to ± 93.4 pmol mol ^−1^ at 19.8 °C and from ± 26.1 to ± 48.9 pmol mol ^−1^ at 25.0 °C. The large uncertainty at 19.8 °C indicates that the corresponding Γ _COS_ value is statistically indistinguishable from zero. By contrast, the Γ _COS_ measured at 25 °C remains significant even when this propagated and instrumental uncertainty is considered. Even accounting for the reported QCLS uncertainty of approximately 7.5 pmol mol ^−1^ for COS (Kooijmans et al., 2016), the Γ _COS_ value at 25 °C remains statistically robust.

 I have not finalized the review of the end of the manuscript but I see that Figs 7-9 and most Tables except Table 1 could be removed as they show results from the biochemical model optimization that, to me, should be removed because I do not see what it brings to the study. My suggestions would make the paper much more digest and strong, and the main message should stay almost unchanged. I hope the authors will agree with this suggestion.

 We appreciate the reviewer’s careful reading and constructive feedback. We understand the concern that the inclusion of biochemical model optimization results may appear to complicate the manuscript. However, we respectfully maintain our vision that the modeling framework is an integral part of the study, serving as a novel diagnostic and interpretive tool rather than the primary result.

 To address the reviewer’s concern about readability and focus, we have taken the following steps:

**1. Clarified the model’s role and rationale in the Introduction** — We have added a short paragraph explaining that the coupled CO₂–COS–H₂O conductance model was developed to mechanistically interpret internal COS processes that cannot be directly inferred from laboratory measurements alone. This emphasizes that the model complements, rather than replaces, the empirical analyses.

**2. Strengthened the Discussion to contextualize the modeling results** — At the beginning of Section 4, we have inserted a paragraph explicitly stating that the model was used to supplement limited observational constraints (e.g., COS compensation points determined at a limited number of temperature levels) and to explore the mechanistic basis of observed COS–CO₂ differences. This addition clarifies the interpretive rather than predictive role of the model. Together, we think that these revisions enhance the manuscript’s coherence and digestibility while preserving its scientific depth. We believe that this integrated experimental–modeling framework is essential to address the study’s main objective: to identify the internal leaf processes governing differential COS and CO₂ flux responses.

 Disentangling these hypotheses using field measurements is challenging due to interrelated and simultaneous variations of environmental conditions (e.g. T _leaf_ , VPD, and g_sw ). Laboratory experiments offer the advantage of observing COS and CO _2_ uptake changes at a leaf level under controlled conditions while independently varying environmental factors. To complement the experimental analysis and mechanistically interpret the observed gas-exchange responses, we developed a coupled CO _2_–COS–H _2_O conductance model based on a leaf conductance model previously established for GPP tracers such as the ∆ ^17^O and ∆ _47_ isotopic composition of CO _2_ (Adnew et al., 2020; Adnew et al., 2021; Adnew et al., 2023). This model serves as a diagnostic tool that enables joint optimization of gas-exchange parameters through the shared stomatal pathway for the three gases and allows inference of internal variables, such as intercellular and chloroplast COS mole fractions, that are not directly measurable.

**<4. Discussion>**

 This study aimed to investigate how leaf COS and CO _2_ fluxes respond differently to environmental changes and to identify which internal processes within leaves govern these differences. Laboratory experiments were conducted under controlled conditions to isolate the effects of stomatal conductance and temperature. However, to provide more insights in the internal processes that control COS exchange, we developed and optimized a coupled CO _2_–COS–H _2_O conductance model to mechanistically interpret the measurement data. Although the model involves some simplified representations of internal processes, these simplifications are minor and do not affect the overall interpretation. In this section, we discuss the uncertainties in both observations and model representations, the physiological implications of the observed COS compensation points, and their broader significance for biochemical parameters and the global COS budget.****
